# Sewage loading and microbial risk in urban waters of the Great Lakes

**DOI:** 10.1525/elementa.301

**Published:** 2018-06-20

**Authors:** Sandra L. McLellan, Elizabeth P. Sauer, Steve R. Corsi, Melinda J. Bootsma, Alexandria B. Boehm, Susan K. Spencer, Mark A. Borchardt

**Affiliations:** * School of Freshwater Sciences, University of Wisconsin-Milwaukee, Milwaukee, Wisconsin, US; † United States Geological Survey, Middleton, Wisconsin, US; ‡ Department of Civil Engineering, Stanford University, Palo Alto, California, US; § United States Department of Agriculture – Agricultural Research Service, Marshfield, Wisconsin, US

**Keywords:** Sewage, Fecal indicator bacteria, Human associated indicators, Urban water systems, Quantitative microbial risk assessment

## Abstract

Despite modern sewer system infrastructure, the release of sewage from deteriorating pipes and sewer overflows is a major water pollution problem in US cities, particularly in coastal watersheds that are highly developed with large human populations. We quantified fecal pollution sources and loads entering Lake Michigan from a large watershed of mixed land use using host-associated indicators. Wastewater treatment plant influent had stable concentrations of human *Bacteroides* and human *Lachnospiraceae* with geometric mean concentrations of 2.77 × 10^7^ and 5.94 × 10^7^ copy number (by quantitative PCR) per 100 ml, respectively. Human-associated indicator levels were four orders of magnitude higher than norovirus concentrations, suggesting that these human-associated bacteria could be sensitive indicators of pathogen risk. Norovirus concentrations in these same samples were used in calculations for quantitative microbial risk assessment. Assuming a typical recreational exposure to untreated sewage in water, concentrations of 7,800 copy number of human *Bacteroides* per 100 mL or 14,000 copy number of human *Lachnospiraceae* per 100 mL corresponded to an illness risk of 0.03. These levels were exceeded in estuarine waters during storm events with greater than 5 cm of rainfall. Following overflows from combined sewer systems (which must accommodate both sewage and stormwater), concentrations were 10-fold higher than under rainfall conditions. Automated high frequency sampling allowed for loads of human-associated markers to be determined, which could then be related back to equivalent volumes of untreated sewage that were released. Evidence of sewage contamination decreased as ruminant-associated indicators increased approximately one day post-storm, demonstrating the delayed impact of upstream agricultural sources on the estuary. These results demonstrate that urban areas are a diffuse source of sewage contamination to urban waters and that storm-driven release of sewage, particularly when sewage overflows occur, creates a serious though transient human health risk.

## Background

Impairment of rivers and beaches by fecal pollution is an ongoing problem for water resource managers and threatens ecosystems and human health worldwide (Garbossa et al., 2017; Reeves et al., 2004; Reischer et al., 2013; Stewart et al., 2008; USEPA, 2009). Urban coastal regions are especially stressed by fecal pollution from watershed sources that can include upstream agricultural runoff and densely populated urban areas near estuaries (Paul and Meyer, 2001; Rothenberger et al., 2009; Sidhu et al., 2013). In the US, more than 50% of the population lives in counties within coastal watersheds, highlighting the disproportionate anthropogenic stress on these watersheds (NOAA, 2013a). This trend is mirrored worldwide with more than 1 billion people living within 50 km of a coast (Kummu et al., 2016). The freshwater coasts of the Great Lakes are particularly sensitive to fecal pollution impacts as these bodies of water serve as drinking water sources to more than 40 million people in the US and Canada (NOAA, 2013b).

Aging sewer infrastructure increasingly threatens water quality as the gap between investments and deterioration widens. An estimated amount of almost one trillion dollars is needed for capital investments to assure the integrity of wastewater infrastructure in the US (USEPA, 2007). The American Society of Civil Engineers ranks the nation’s wastewater conveyance and treatment systems as a D+ (ASCE, 2017). Sewage has been detected in urban rivers and coastal waters in the absence of recognized sewage overflows (Sercu et al., 2009; Wiegner et al., 2017). Sewage can enter surface waters in a number of different ways. It can leak from deteriorating pipes into the surrounding ground and subsequently infiltrate stormwater systems or groundwater, which can then transport contamination to rivers and beaches. Sewage overflows continue to be a major problem in older cities in the Midwest, Northeast, and Pacific Northwest, where nearly 860 communities have combined sewer systems (USEPA, 2004), where both stormwater and sanitary sewage are captured in the same system of pipes. These systems become inundated with stormwater and overflow to rivers, resulting in the release of 850 billion gallons of untreated sewage mixed with stormwater each year (USEPA, 2004). A total of 184 of these systems discharge to the Great Lakes drinking water sources (USEPA, 2016). With the number of extreme storms expected to increase in areas of the US that have a high density of combined sewer systems, combined sewer overflows (CSOs) may rise, threatening drinking water sources (Patz et al., 2008; Trtanj et al., 2016).

To track sewage pollution, water quality indicators more specific than those used traditionally are needed (McLellan and Eren, 2014). For more than 100 years, rivers and streams have been monitored for waterborne pathogen risk using fecal organisms that are easy to culture, including fecal coliforms, *E. coli* and enterococci. However, human sources cannot be distinguished from domestic pet, wildlife or agricultural animal waste, because all of these hosts carry *E. coli* in their guts. Using new indicators that target fecal anaerobic bacteria (Fiksdal et al., 1985; Eren et al., 2015) specifically associated with humans can provide evidence of sewage contamination in waterways. Identifying and remediating sources of human fecal pollution is important, because they have a higher probability of carrying human pathogens, especially human viruses, compared to many animal sources (Schoen et al., 2011). Agricultural runoff can also present a high health risk from certain bacterial and protozoan pathogens such as *Salmonella*, *E. coli* O157:H7, and *Cryptosporidium* (Ferguson et al., 1996; Medema et al., 1997; Chekabab et al., 2013).

In this study, our goal was to couple intensive sampling before, during, and after storm events with measurements of both traditional fecal indicators and host-associated indicators to quantify sources of fecal pollution. Our work sheds light on the causes of chronic fecal contamination observed in an estuary, and attempts to benchmark quantitatively the amount of sewage being released from a highly urbanized watershed following rainfall. Concurrent pathogen measurements in sewage allowed us to relate human-associated indicator levels to relative risk of illness from pathogen exposure. Quantifying sources of agricultural and sewage fecal pollution across the hydro-graph illustrates the dynamic and complex contamination inputs from watershed sources.

## Methods

### Area of study and sampling.

**Figure 1:**
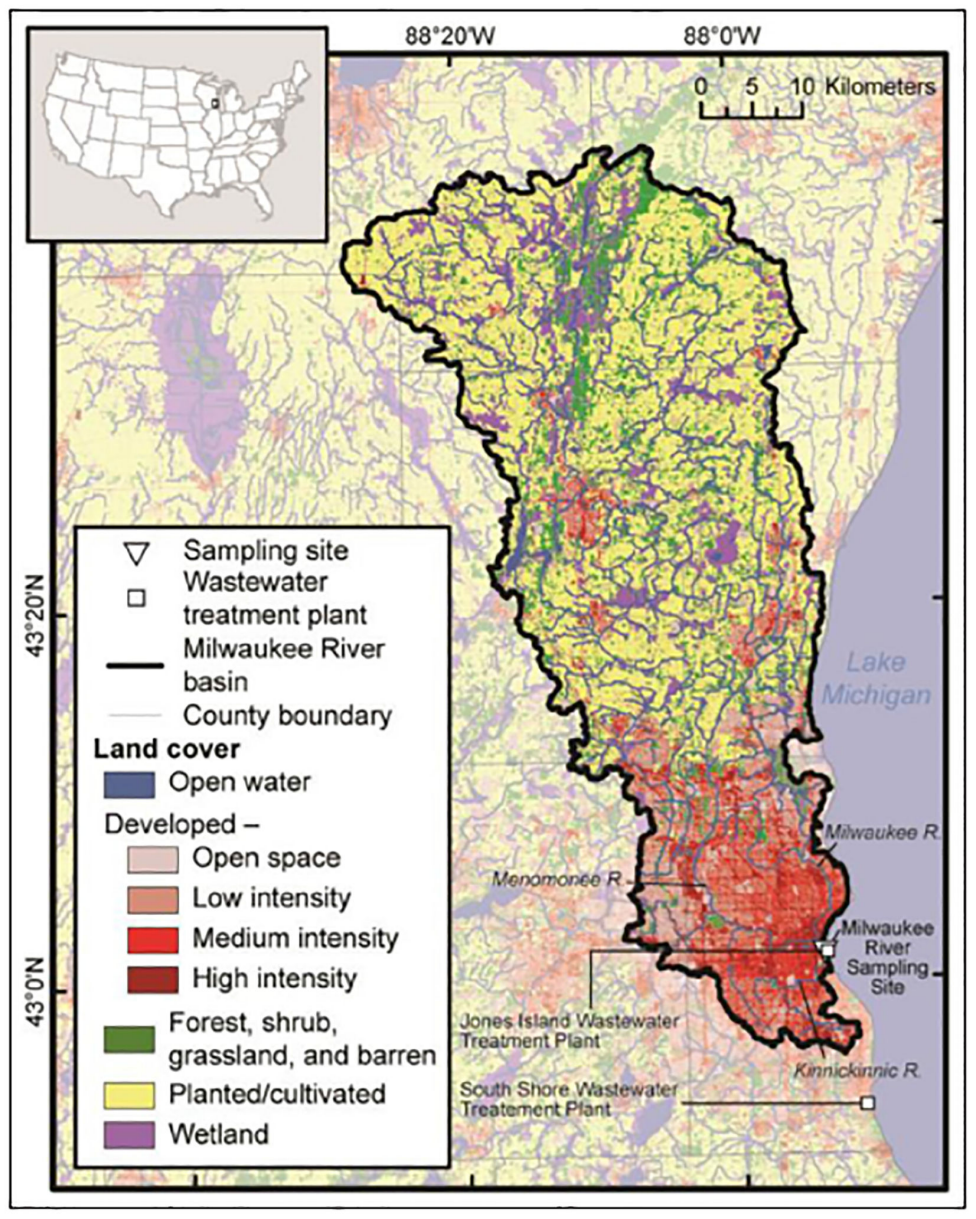
The Milwaukee River basin as the study site. Land cover, the three main rivers draining to the estuary, and two wastewater treatment plants are labeled. Map is comprised of various spatial datasets: state boundaries (Instituto Nacional de Estadística Geografía e Informática et al., 2006), county boundaries (National Atlas of the United States, 2005), hydrography (U.S. Environmental Protection Agency and U.S. Geological Survey, 2005), land cover (Fry et al., 2011), and watershed boundaries (modified from Southeastern Wisconsin Regional Planning Commission, Environmental Division and GIS Division, 2005). DOI: https://doi.org/10.1525/elementa.301.f1

The Milwaukee River Basin encompasses 2280 km^2^ of mixed land use with three main rivers that converge in the Milwaukee estuary within 0.5 km of the Milwaukee harbor, which is located along the western shore of Lake Michigan. The study site is shown in Figure 1. This basin is typical of coastal watersheds, with the largest river, the Milwaukee (MKE) River, receiving drainage (1813 km^2^) from upstream agricultural land that includes dairy farms, suburban areas, and highly developed areas of several small communities and the northern parts of metropolitan Milwaukee. The Menomonee (MN) River receives drainage (352 km^2^) from primarily suburban and urban areas. The Kinnickinnic (KK) River drains the smallest watershed of 65 km^2^ and is highly urbanized. There are approximately 190 combined sewer outfalls in the lower reaches of these rivers that discharge during extreme rain events. Two wastewater treatment plants (WWTPs) service this area: treated effluent is discharged from Jones Island WWTP to the Milwaukee harbor, adjacent to the estuary and below our sampling site; and South Shore WWTP discharges to Lake Michigan approximately 15 km south of the Milwaukee harbor.

A portable automated sequential sampler (3700 full size, Teledyne ISCO, Lincoln, NE) was used to collect hourly composite samples (15-min time-weighted subsamples in a single 1-L bottle) downstream from the confluence of the MKE, MN, and KK rivers in the Milwaukee estuary. Samples were collected before, during, and after rain events from April to October during 2009, 2010, and 2011. The sampler was packed with ice at the start of sampling and replenished with ice each day. Samples were analyzed for *E. coli* and enterococci by culture as described below. Human-associated indicators were analyzed by quantitative polymerase chain reaction (qPCR) in the 1-h samples, or composites of these samples representing 2-h or 4-h time frames. For this analysis, sample volumes of 200 mL were filtered onto a 0.22 μm mixed cellulose ester filter (47-mm diameter; Millipore, Billerica, MA) and filters were stored at −80°C until processed for DNA extraction. Analysis of the traditional culturable indicators, *E. coli* and enterococci, and of qPCR genetic markers in the 1-h samples demonstrated that 2-h and 4-h composites captured the changes in concentrations similar to what was measured in the individual 1-h samples. Table S1 shows the event types, dates and number of samples analyzed by culturing and qPCR.

### WWTP influent samples.

Untreated sewage influent samples were collected as 24-h flow-weighted samples from a single day, or were combined as seven-day composites. Samples were collected by the Milwaukee Metropolitan Sewerage District as part of their daily monitoring program two to three times per month over a two-year period (n = 98). Seven-day composites were held at 4°C prior to processing within 48 h of the end of collection. Single-day samples were processed the same day as collection. A volume of 50 mL was filtered onto a 0.22 μm mixed cellulose ester filter (47-mm diameter; Millipore, Billerica, MA), and the filters were stored at −80°C until processed for DNA extraction. Composite and single-day samples were analyzed for human *Bacteroides* (HB) and human *Lachnospiraceae* (Lachno2), as well as the traditional indicators, *E. coli* and enterococci, by qPCR as described below. Culturing of traditional indicators was not performed on these samples. Data were log-transformed, and geomean concentrations and standard deviations of the geomean were calculated.

Norovirus concentrations were determined in these same samples, using 2 L of sample, as part of another study. Text S1 details the methods for GI and GII noroviruses, which were used for risk assessment calculations. Data for norovirus concentrations are deposited in the U.S. Geological Survey (USGS) National Water Information System Web Interface and can be retrieved by USGS parameter codes, parameter names, and microbiological category. For GI norovirus, parameter code is 31765, parameter name is “Norovirus genogroup I, qPCR”, and microbiology category is “Human Virus”. For GII norovirus, parameter code is 31766, parameter name is “Norovirus genogroup II, qPCR”, and microbiology category is “Human Virus”.

### Traditional and alternative indicator analysis.

Estuarine samples and selected WWTP influent samples were analyzed for culturable *E. coli* and enterococci using USEPA methods by filtering the appropriate volume to obtain a countable density of colonies, 0 to 220 colonies per plate (USEPA, 2002, 2006). Samples were also analyzed for *E. coli*, enterococci, and host-associated markers by qPCR as described previously (Sauer et al., 2011; Templar et al., 2016). Briefly, DNA was extracted from stored filters using MPBIO FastDNA® SPIN Kit for Soil (MP Biomedicals, Santa Anna, CA). Samples were generally analyzed within six to nine months after collection, with the exception of samples from 2009 and 2010 which were analyzed for Lachno2 in 2011 as the assay was developed. These latter samples were reanalyzed for HB, and no significant difference was detected between HB values determined after <9 months of storage and those determined within 12–30 months of storage (p < 0.05).

DNA extraction efficiency was tested in a subset of river water samples (n = 20) using salmon testes DNA (Sigma, catalog# D1626) according to USEPA Method 1611 (USEPA, 2012a). This method involved adding 0.2 μg of salmon testes DNA to 1 mL DNA extraction buffer and extracting a blank filter (n = 5), followed by qPCR analysis of the extracted samples. The mean (± standard deviation) extraction efficiency was 19.8% (±6%), which was similar to the efficiency we determined previously (15.3 ± 2.7%; Sauer et al., 2011). When the salmon testes DNA was added to river water sample filters (n = 20), extraction efficiencies increased to an average of 46.5% (±3%), suggesting that sample DNA acts as a carrier for the low amounts of spiked DNA. Inhibition of the PCR was tested independently of extraction in a subset (n = 20) of river water samples by adding salmon testes DNA directly to extracted river water samples to a final concentration of 0.2 ng μL^−1^ in each sample. DNA concentrations, quantified as described in Method 1611 (USEPA, 2012a), ranged from 84 to 105% of the expected concentrations, with an average of 95% (±7%), indicating no inhibition. As these results are consistent with other studies of stormwater samples, where no inhibition was noted using internal amplification controls consisting of synthesized plasmids with an unrelated target sequence (Sauer et al., 2011), we did not test for inhibition in individual samples.

Assays for *E. coli* (Sauer et al., 2011), enterococci (USEPA, 2012a), HB (Templar et al., 2016), Lachno2 (Newton et al., 2011; Templar et al., 2016), and ruminant markers (Reischer et al., 2006) have been described previously. An Applied Biosystems StepOne Plus™ system with Taqman chemistry (Applied Biosystems; Foster City, CA) was used for qPCR. Reactions were carried out in volumes of 25 μL, with 5 μL of sample added as template, using Taqman® Gene Expression Mastermix kit according to manufactures instructions (Applied Biosystems, Foster City, CA). Amplification products cloned into TA vector 2.1 (Invitrogen, Carlsbad, CA) were used as standards. Standard curves were determined using a range of 1.5 × 10^6^ to 1.5 copy number (CN) per reaction. The lower limit of quantification was determined to be 15 CN per reaction, which is equivalent to 225 CN per 100-mL sample considering a volume of 200 mL was filtered for each sample and extracted DNA was eluted in a volume of 150 μL. Signals below 35 cycles (and not within the quantifiable range) were considered detectable but not quantifiable. All qPCR runs included two previously analyzed environmental samples as controls. All no-DNA template controls were negative. All assays were performed in duplicate and compared to values in standard curves. Assay primers and standard curves are reported in Table S2.

### Quantitative microbial risk assessment (QMRA).

We predicted the risk of enteric illness associated with recreational exposure to human markers from sewage in the river water when the concentrations were 1, 10, 10^2^, 10^3^, 10^4^, and 10^5^ CN per 100 mL in river water. To do so, we modeled the distributions of HB, Lachno2, and norovirus measured in untreated sewage using the 98 WWTP influent samples described above. We combined the data from the two WWTPs and modeled them as log_10_-normal with mean and standard deviation.

To calculate the risk, we followed the methods described in detail by Boehm et al. (2015). In brief, we started with the assumed concentration of human marker in river water (1, 10, 10^2^, 10^3^, 10^4^, or 10^5^ CN per 100 mL in river water). We then used a Monte Carlo approach to randomly draw from the distribution describing the human marker concentration in sewage, and used this value to calculate the fraction of sewage present in a volume of river water. Subsequently, we drew a concentration of norovirus in sewage from its distribution to calculate the concentration of norovirus present in the river water. The volume of water ingested during swimming was assumed to follow the ln-normal distribution reported by Dufour et al. (2006). A number was drawn from this distribution and, along with the concentration of norovirus present in river water, used to calculate the dose a swimmer consumes of norovirus. The norovirus dose-response curve was used to determine the probability of infection given that dose, and the probability of illness given infection was assumed to be 0.6 (Teunis et al., 2008; Boehm et al., 2015). This procedure was repeated 10,000 times for each human marker concentration in river water, and the process was completed for each of the two human markers (HB and Lachno2). The end result is a distribution of predicted illnesses per 100 swimmers for each concentration of human marker. The QMRA approach used herein differs from that described by Boehm et al. (2015), because norovirus is the only pathogen considered in the QMRA. We took this approach because norovirus contributes most of the risk in recreational water QMRAs that have modeled effects of sewage exposure on illness (Soller et al., 2010a; Boehm et al., 2015). All model calculations were carried out in Matlab (Natick, MA).

### Hydrology, flux, and load calculations.

The USGS gauging station at Jones Island WWTP in Milwaukee, Wisconsin, provided flow data for the MKE, MN, and KK rivers, and the estuary. The 24-h mean peak concentrations for HB and Lachno2 genetic markers were calculated for each event in Microsoft Excel version 15.64 using a moving average. Loads were calculated for selected events that included the rising and falling limb of the hydrograph by using 5-min instantaneous flow with interpolation of HB and Lachno2 concentrations from 2-h measurements.

### Statistics.

Data were log-transformed to calculate the geometric mean and standard deviation. Students T-test was used to compare human marker concentrations in composite and single-day samples, and at the two WWTPs, and to compare ratios of indicators in different sample types. Pearson correlations were conducted on log-transformed data to evaluate the relationship between the two human markers in sewage and in water samples.

### Data submission.

Data have been archived using Dash (University of California Curation Center) in the DataOne Project under the title of this manuscript.

## Results

### Stability of human *Bacteroides* (HB) and human *Lachnospiraceae* (Lachno2) genetic markers in untreated sewage.

Sewage influent samples (n = 98) from two separate WWTPs were collected between May 2009 and April 2011 and analyzed for two human-associated markers, HB and Lachno2, and the traditional indicators, *E. coli* and enterococci, by qPCR. The human marker concentrations were high in sewage influent and very consistent between the two WWTPs (Table 1). While there was variability among sample days, concentrations were generally within the same order of magnitude, with geomean concentrations of 2.77 × 10^7^ CN 100 mL^−1^ for HB and 5.94 × 10^7^ CN 100 mL^−1^ for Lachno2. No significant difference was detected between treatment plants, or between single and composite samples, with the exception of Lachno2, which was significantly higher in single samples from South Shore WWTP compared to composite samples from the same plant or to Jones Island WWTP single or composite samples (Table 1). The median ratio between Lachno2 and HB was 2.37. The qPCR analysis of traditional indicators demonstrated that *E. coli* was lower than the human-associated markers by more than an order of magnitude and that enterococci were generally higher than both human markers. In general, however, all indicator levels were very high in untreated influent, with about 10^6^ CN 100 mL^−1^ for *E. coli*, 5 × 10^7^ CN 100 mL^−1^ for human markers, and 10^8^ CN 100 mL^−1^ for enterococci.

### Concentrations of human viruses in sewage and risk relationship to indicators.

Human viruses were measured in 70 of the 98 untreated WWTP samples as part of another study (Lenaker et al., 2017). Detection of viruses was intermittent and levels considerably lower than the human-associated bacterial markers. Norovirus GI was detected in 30 of the 70 samples at concentrations of genomic copies (GC) that varied over three orders of magnitude. The maximum concentration was 1.83 × 10^6^ GC L^−1^, with a geomean concentration of 3.18 × 10^4^ GC L^−1^ for the positive samples. Norovirus was detected in every month except February. Genotype GII was not detected in any of the samples. In general, the frequency of detection was higher from spring to early fall, but norovirus concentrations were highest in January. The full dataset for human viruses is available in the USGS National Water Information System Web Interface (as referenced in Methods).

**Figure 2:**
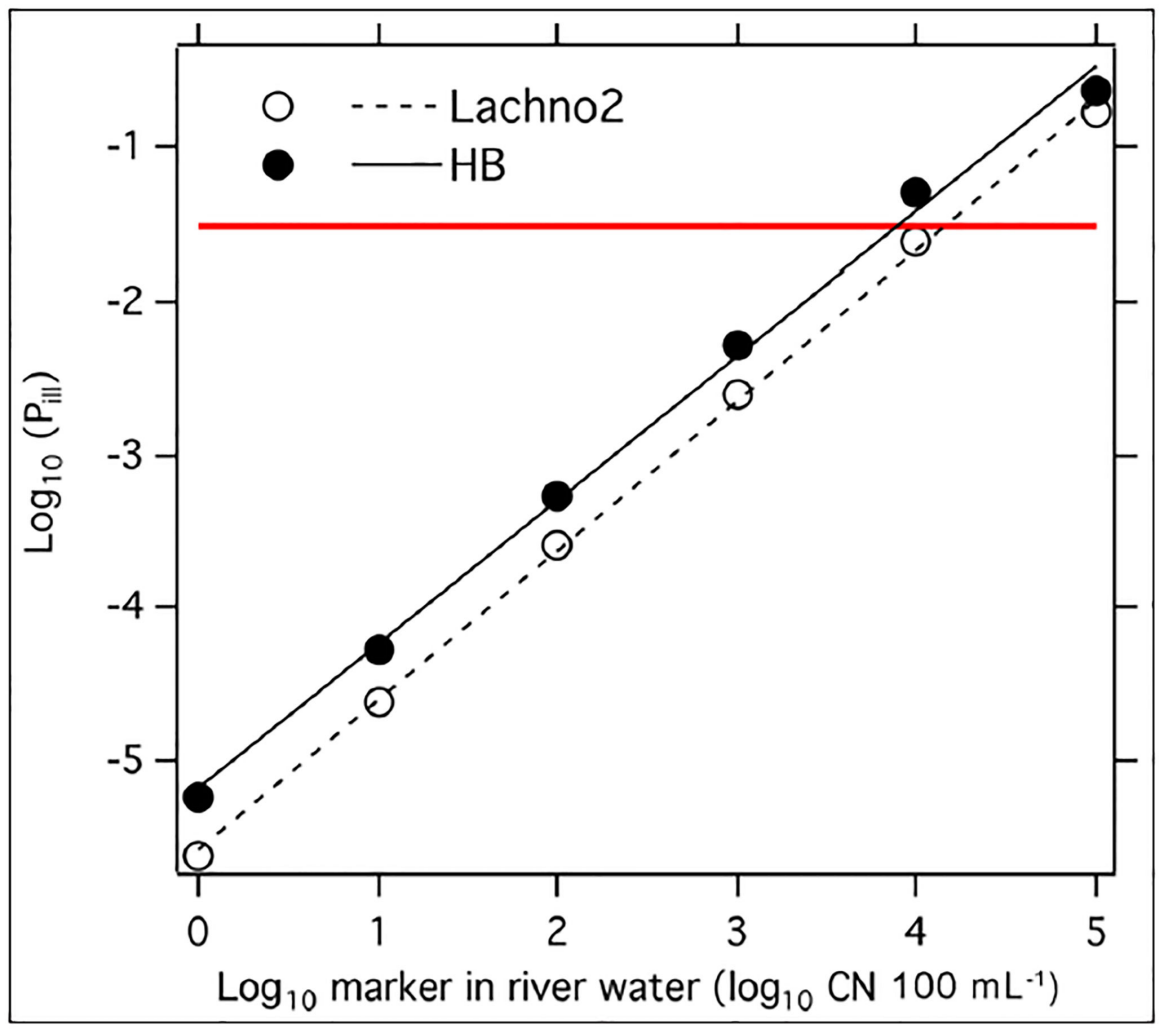
Median illness risk as a function of log_10_-transformed concentration of HB and Lachno2. The median probability of illness (P_ill_) from the simulated P_ill_ distributions for each considered marker concentration is shown as a symbol. A linear least squares approach was used to generate a linear curve fit between log_10_-transformed median P_ill_ (y) and log_10_-transformed marker concentration (x). The equations for the lines are for Lachno2: y = 0.976 × −5.579 (R^2^ = 0.999), and for HB: y = 0.941 × −5.185 (R^2^ = 0.997). The threshold of 30 per 1,000 illnesses (3%, shown as a red horizontal line) crosses the two curve fit lines at 14,000 copy number (CN) 100 ml^−1^ water (Lachno2) and 7, 800 CN 100 ml^−1^ river water (HB). DOI: https://doi.org/10.1525/elementa.301.f2

A QMRA was used to estimate the risk expected from exposure to raw sewage in river water. We used observed concentrations of HB, Lachno2, and norovirus GI in untreated sewage to construct log_10_-normal distributions (Table S3). These distributions were used as inputs to the QMRA. Distributions of risk obtained from the 10,000 simulations for each indicator concentration are shown as box and whisker plots in Figures S1 and S2. The median values for risk of illness for each concentration of human-associated indicators that was generated in the simulation are plotted in Figure 2.

### Quantitative measurements of human *Bacteroides* and *Lachnospiraceae* signals during known sewage contamination events in the environment.

We used two known CSOs to evaluate how well our human-associated genetic markers measured sewage contamination quantitatively. We assessed the human fecal pollution signals in the estuary before, during, and after two storm events with CSOs of different magnitudes. The rain event of 19 June 2009 was accompanied by a large CSO approximately five times the magnitude in volume and duration of a smaller CSO that occurred during the 20 June 2011 rain event (Table 2). The magnitude difference in volume of combined sewage was mirrored in the differences in mean concentrations during the event, as well as the overall load of human indicators into the lake (Table 2). The mean concentration of the HB marker was 5.7-fold higher, and the HB load 6.2-fold higher, during the large CSO compared to the smaller event. In addition to reported CSO volumes, upstream communities also reported sanitary sewage overflows (SSOs) for both events, which would be expected to increase the amount of untreated sewage in the estuary during the same time frame. Lachno2 in sewage was highly correlated to HB (r = 0.96) across both CSO events.

Using the geomean of HB concentrations in untreated sewage, we estimated that the discharge from the estuary to the lake was comprised of 0.81% and 0.14% untreated sewage for the large and the small events, respectively. From the loads measured in the estuary (assuming that all sewage was from the CSO) and the total volume of CSO release reported, we estimated that the larger CSO was comprised of 2.76% untreated sewage mixed with stormwater in the actual CSO release. Estimates from the smaller event suggested that the CSO discharge was slightly more concentrated, with 3.14% untreated sewage mixed with stormwater.

In the 24-h period following the larger CSO, mean concentrations of the HB marker dropped an order of magnitude to 2.71 × 10^4^. The smaller event followed a similar pattern, but for a sharp increase in HB concentrations 14 h after the end of the CSO. This second peak in HB concentrations declined to <10^3^ within the next 16 h, and may have corresponded to SSO discharges from communities upstream that extended past the period of CSO discharge. A third CSO event was partially sampled, with samples taken 18 h after the start of the event and again one day after the event (Table 3). This storm event resulted in greater than 20 cm of rain in the watershed in a 24-h period and was accompanied by widespread flooding. Due to lack of access to the sampling site, the peak concentrations were likely missed. These results indicate that human-associated indicators coupled with hydrological measurements and high frequency sampling can be used to estimate the quantity of untreated sewage entering water bodies.

### Sources of fecal pollution during baseflow and rain events.

**Figure 3:**
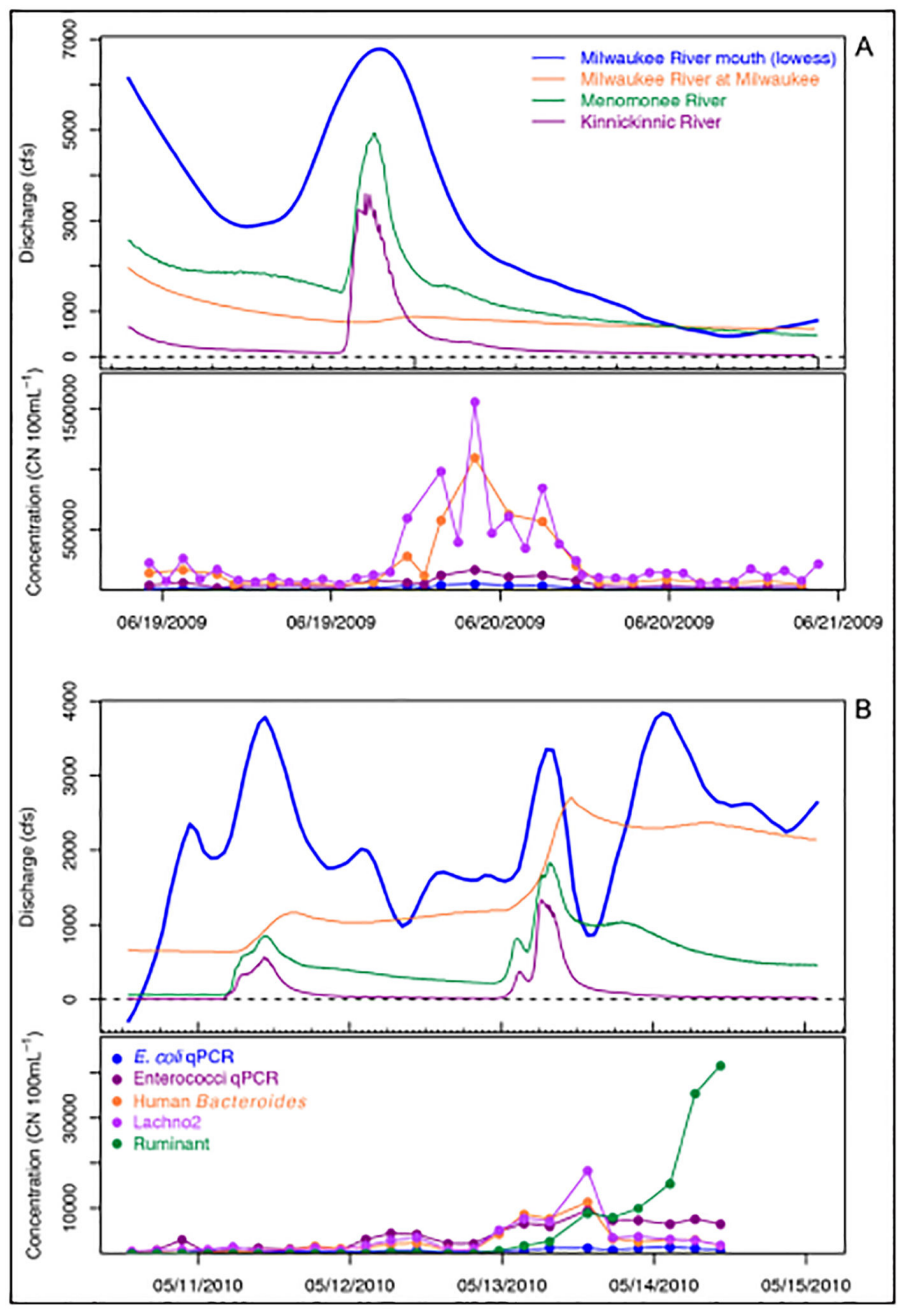
Source-specific indicators (human) and general indicators (*E. coli* and enterococci) measured in the estuary. Samples were collected across the hydrograph during the CSO on 20 June 2009 **(A)** and the rain event on 15 May 2010 **(B)**. Human fecal indicators, human *Bacteroides* and *Lachnospiraceae* (Lachno2), were detected at high concentrations following the peak of the discharge for the two urban rivers, the Menomonee and Kininnickinnic rivers. Hydrographs from all events are shown for culture and qPCR in Figures S3–13 and Figures S14–24, respectively. Lowess indicates locally weighted regression using the lowess function in R (R core team, 2017). Dates are day/month/year. DOI: https://doi.org/10.1525/elementa.301.f3

There was evidence of sewage contamination during baseflow conditions; i.e., when there had been no rain within 24 h. Mean 24-h concentrations were generally low compared to means during rain events (Table 3). During rain events with no reported sewage overflows, the HB genetic marker was detected consistently but at significantly lower concentrations compared to the large CSO event (Figure 3). The full set of hydrographs for events in Table 3 with human-associated indicators and culture data for traditional indicators are presented in Figures S3–S13, and hydrographs with human-associated indicators with qPCR data for traditional indicators are presented in Figures S14–S24. The 24-h peak means of HB (and Lachno2) concentrations varied for different storms, ranging from 1.59 × 10^3^ to 9.72 × 10^4^ CN 100 mL^−1^. All indicator concentrations rose as the MKE, MN, and KK rivers increased in flow. After the peak of the hydrograph in the urban rivers, the human bacterial signal decreased, but the traditional indicators remained elevated. In selected events, we measured the ruminant marker and found that concentrations increased at the estuary approximately one day following the start of the rain event and increased as the human indicators decreased. The ruminant marker was detected in all sampled rain events when the MKE River was a large contributor of flow to the estuary, indicating that agricultural inputs from the upper reaches of the watershed likely impacted estuary and nearshore waters.

### Relationships between *E. coli*, enterococci, and human-associated indicators in untreated sewage and contaminated waters.

**Figure 4:**
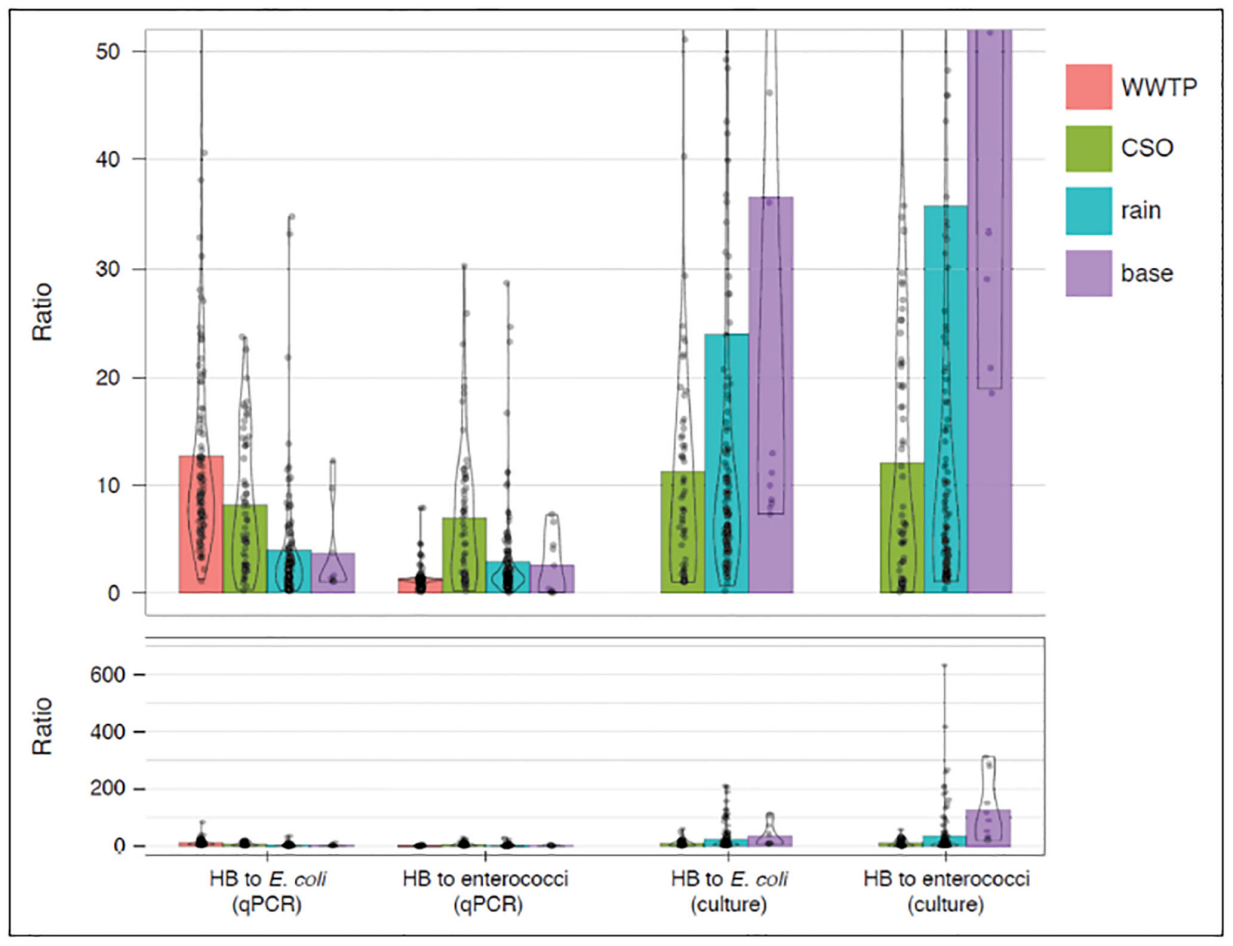
Ratios of HB human marker to traditional indicators measured by qPCR and culture methods. Top panel truncates values above 50 to show detail of lower values. Lower panel shows full range of ratios, which has samples with HB to cultured *E. coli* or enterococci > 50. Sample date 4 June 2010 was excluded from analysis due to extremely high HB levels that were similar to sanitary sewer overflow conditions, but could not be confirmed. The enterococci copy number was divided by four, assuming four copies per cell, before calculating ratios of HB to enterococci by qPCR for visual comparisons with ratios of HB to enterococci by culture. *E. coli* qPCR targets the single-copy *uidA* gene. WWTP indicates wastewater treatment plant; CSO, combined sewer overflow. The width of the outlines for each data series is proportional to the relative density of points. DOI: https://doi.org/10.1525/elementa.301.f4

We compared human-associated indicators with traditional indicators measured by qPCR. During the large CSO event, the HB concentration was 14-fold higher than *E. coli* and 3-fold higher than enterococci. For the event with a lower CSO release volume, the HB concentration was only 5 times higher than *E. coli* and half the concentration of enterococci. These results demonstrate that there is an increasing human signal compared to traditional indicators with known amounts of increasing sewage. However, during rain events with no reported sewage overflow, as well as during baseflow conditions, ratios of HB to *E. coli* and to enterococci were significantly lower than under CSO conditions (p < 0.05) and highly variable across the hydrograph (Figure 4), indicating that a mixture of human and nonhuman sources were present, with human sources dominating for only a short time at the peak of the hydrograph. While the overall trends in these ratios were significant, individual samples had high variability (Figure 4). However, when considering the ratios of HB CN and cultured traditional indicators, we observed a different pattern. Rainfall and baseflow had higher HB CN per cultured cell of *E. coli* or enterococci than CSOs. Further, there was a larger disparity between HB CN and cultured traditional indicators overall compared with the differences between HB and traditional indicators measured by qPCR.

## Discussion

Frequent detection of sewage in estuaries and on beaches (Ferguson et al., 1996; Bower et al., 2005; Korajkic et al., 2011; Johnston et al., 2013; Templar et al., 2016) illustrates the ongoing challenges of maintaining adequate sanitation infrastructure in dense urban areas. Sewer infrastructure networks can comprise more than 10,000 miles of sanitary sewer pipes in a large city. One study reported that up to 4% of sanitary sewer pipes may be at risk for failing (Baah et al., 2015). There are multiple pathways for sewage to escape these systems, including illicit connections or leaking pipes (O’Shea and Field, 1992; Marsalek and Rochfort, 2004). In the latter case, contamination may be mobilized from surrounding soils after rain events and infiltrate ground water (Yau et al., 2014), drinking water (Hunt et al., 2010) and adjacent stormwater systems (Sauer et al., 2011). In cases of heavy rain, systems can become inundated with rainwater and overflow causing CSOs or SSOs. In this study, we have demonstrated that there is a quantifiable pulse of sewage released from an urban area each time it rains, resulting in contamination levels that create a potential public health risk.

### Quantifying sewage inputs from urban areas

We found that HB and Lachno2 were at predictable concentrations in untreated sewage, which offered the opportunity to benchmark environmental contamination against the equivalent volume of untreated sewage. Previous work in our laboratory demonstrated that levels of human fecal bacteria in untreated sewage from 71 different cities across the US were relatively consistent (Newton et al., 2015), and that levels at the Milwaukee study site were stable over a three-year period (McLellan et al., 2013). Human *Bacteroides* as defined by the HF183 genetic marker (Bernhard and Field, 2000) or the HB marker (Sauer et al., 2011) and Lachno2 have been detected widely in field studies (Ahmed et al., 2008; Newton et al., 2011; Jarde et al., 2018). The HF183, HB, and Lachno2 are highly abundant in humans but are not strictly specific, as these markers have been found sporadically in other hosts such as dogs and deer (Boehm et al., 2013; Fisher et al., 2015). Using the two genetic markers in combination is expected to improve reliability, as cross-reacting hosts are unlikely to have both indicators (Fisher et al., 2015; Wang et al., 2010). The consistent occurrence of human-associated fecal indicators across the US suggests that urban areas can be compared to each other, and quantitative water quality criteria could be developed based on new indicators of sewage contamination. In other regions of the world, differences in the human gut microbiome (influenced by diet and other factors) may determine which indicators are most useful (Muegge et al., 2011; Walker et al., 2011; Reischer et al., 2013; Koskey et al., 2014).

### Sewage signals following storm events

High frequency sampling allowed us to quantify mean concentrations over long periods of time (i.e., 24 h or more), and hydrological measurements allowed us to quantify loads. As flow increased in the urban rivers, the concentrations of HB and Lachno2 human markers increased, suggesting rainfall intensities were a driver for transport of sewage into rivers. In rainfall events of greater than 5 cm in 24 or 48 h, there was a disproportionate increase in both peak mean 24-h concentrations and load. In addition, 12 h after the end of a CSO on 11 June 2011, there was an unexpected spike in human-associated indicators, suggesting that during this time of heavy rain unrecognized overflows in the sanitary sewage systems upstream might have contributed additional sewage. These results suggest that there may be a critical threshold for conveyance systems within a city. Establishing the sensitivity of a city to rainfall events could enable warning systems to advise the public to avoid risk of exposure, as well as help guide investments to improve capacity, particularly under changing climate conditions where storm events of increased intensity are predicted to increase in the northeast and Great Lakes regions of the US (USEPA, 2008; Trtanj et al., 2016).

### Comparison of traditional and human-associated indicators

We examined patterns in the ratios of HB to traditional indicators over multiple hydrological conditions. Most notably, during rain events the ratios of HB to *E. coli* in river samples measured by qPCR were lower than in WWTP influent, which suggests that other fecal sources contributed *E. coli*, but not human markers, to the contaminated river water. These differences were illustrated in our high resolution sampling over the hydrograph, where ruminant sources dominated during the second half of the storm without a parallel drop in general indicators (Figures 3 and S3–S13).

The differences in indicators measured by qPCR (i.e., detection of intact cells) and culture (i.e., detection of viable cells) might provide clues as to the age of pollution. Microcosm studies have suggested that qPCR markers from different organisms are lost at a similar rate (Mattioli et al., 2017). However, decay of cultured indicators are expected to be more rapid than loss of qPCR signal. Differential decay can be influenced by temperature and sunlight (Korajkic et al., 2014; Maraccini et al., 2016), and sunlight has been shown to influence decay of viable cells more than molecular targets (Boehm et al., 2009; Green et al., 2011). Our results are consistent with this concept, as ratios of HB to cultured *E. coli* were greater than ratios of HB to qPCR-based *E. coli*. We also noted that recent pollution (i.e., from a CSO) had a smaller ratio of HB CN to cultured *E. coli* or enterococci than when pollution may have been in the environment longer (i.e., rain or base-flow samples). Multiple factors can influence the ratios of a host-associated indicator to traditional indicators in a single sample, including inputs from multiple sources, timing of human and nonhuman inputs, and differential decay of signals over time. As a result, the relationship between human-associated and traditional indicators cannot be interpreted in single samples; however, ratios may reflect a general trend in the system.

### Human-associated indicators of pathogens and waterborne illness risk

Human markers were three to four orders of magnitude higher than virus concentrations in untreated sewage (Table 1), suggesting that human-associated indicators might be useful for estimating risk in surface waters with dilute amounts of sewage contamination. Pathogens are technically difficult and expensive to measure in the environment, and are often present at low levels (Gentry-Shields et al., 2012; Corsi et al., 2014); in cases where pathogen test results are negative, the pathogens could be present but below detection limit. In work conducted concurrently with the present study, viruses were sampled at the estuary during one of the CSO events (Lenaker et al., 2017); however, no viruses were recovered, despite known sewage contamination. Because virus occurrence in the human population is seasonal (Sedmak et al., 2005), the exposure pathway can be characterized by using genetic markers that are relatively stable in sewage (the primary reservoir for human waterborne pathogens), without depending on capturing virus occurrence at the sampling time.

The QMRA model indicates that we can expect greater than a 10% illness risk for swimmers exposed to river water after heavy rain in the absence of sewage overflows, based on the concentrations of HB and Lachno2 that we measured. Risk from CSO-contaminated water exceeded the upper bound of 10% in this analysis. The model results could be used to derive risk-based thresholds for these two indicators, which could be defined as the concentration at which the median simulated risk is 0.03, similar to the USEPA benchmark risk used for establishing recreational water quality criteria (USEPA, 2012b). Our estimates were very similar to what was reported in Boehm et al. (2015) for HF183 (4,200 CN 100 mL^−1^), despite the two studies using different datasets for the human *Bacteroides* marker in untreated sewage. In this study, norovirus was measured in the same samples as the HB marker, whereas Boehm et al. (2015) used previously published concentrations for norovirus that were independent of the samples in which the HF183 marker was measured. The human *Bacteroides* HB and HF183 assays target the same organism, and both employ the HF183 primer, but utilize slightly different reverse primers and probes (Templar et al., 2016).

Recent research estimates that nearly 90 million incidences of waterborne disease occur annually in the US due to recreational water exposure (DeFlorio-Barker et al., 2018). Moving forward, the risk-based thresholds we have developed could be particularly valuable for understanding human health risks associated with recreational contact, as they offer a stronger scientific basis for inferring risk than general indicators. Estuaries and harbors are generally not used for swimming; however, kayaking, rowing, and other recreational activities are becoming more popular. Direct exposure would most likely occur from mishaps, when individuals become submerged in the water. Of higher concern may be urban beaches near harbors and river outlets (Wiegner et al., 2017). In these cases, hydrodynamic models or other predictive tools would be useful to further estimate the amount of river water delivered to a beach site. Future work should examine whether aging of the contamination affects the estimation of threshold levels, as they were derived in this study for the specific scenario where the sewage contamination is very recent. The differential survival and transport of viruses and bacteria may further affect risk assessment relationships that employ bacterial indicators. Directly measuring pathogens, or surrogate viruses common to humans, in high volumes of the river or beach water under known contamination conditions could be the next step towards developing site-specific criteria. While norovirus was detected throughout the year in this study, concentrations were higher in winter than summer; therefore, next steps towards evaluating risk could incorporate a seasonal component into risk modeling.

### Evaluating and managing urban waters

In the US, the current method for responding to impaired waters is to implement total maximum daily load (TMDL) regulations. In this framework, fecal coliforms are targeted to reduce risk from pathogens. However, different sources of fecal coliforms do not carry the same risk (Soller et al., 2010b). Stormwater-impacted rivers have shown evidence of sewage contamination with only a modest correlation to fecal coliforms (Sauer et al., 2011; Templar et al., 2016), suggesting that both human and nonhuman sources were present. Many watersheds have upstream agricultural land use, which can contribute fecal pollution from agricultural animal sources, as well as from leaking septic systems (Verhougstraete et al., 2015). The presence of traditional indicators in the absence of a sewage or agricultural signal demonstrates that stormwater carries non-point pollution from urban wildlife and domestic pets, contributing fecal coliforms to waterways that are not considered as serious a health risk as sewage (Soller et al., 2010b). To effectively address pathogens, TMDL regulations need to be developed with an understanding of the source of contamination (He et al., 2007).

Urban waters are economic drivers in cities and are used increasingly used for recreation. Higher resolution methods for identifying sewage sources could offer information on areas where pathogens are more likely to occur, and could provide evidence of potential risk. Here we quantified on a watershed scale the amount of sewage released from an urban area, creating risk to humans and the ecosystem. The ability to track this contamination using newly developed host-associated indicators advances our ability to assess and manage freshwater resources.

## Supplementary Material

Supplemental Fig S14-S24

Supplemental Fig S3-S13

Supplemental Text_Tables_Fig S1-S2

## Figures and Tables

**Table 1:** Geomean concentrations of indicators and norovirus in WWTP samples collected over a two-year period, 2009–2011. DOI: https://doi.org/10.1525/elementa.301.t1

Site	Type of sample^a^	n	HB (CN L^−1^)^b^	Lachno2 (CN L^−1^)^b^	Enterococci (CN L^−1^)	*E. coli* (CN L^−1^)	G1 norovirus (GC L^−1^)^c^
Jones Island WWTP	Composite	33	2.76 × 10^8^	5.36 × 10^8^	1.38 × 10^9^	2.22 × 10^7^	2.85 × 10^4^
	Single	16	2.08 × 10^8^	6.30 × 10^8^	8.75 × 10^8^	3.19 × 10^7^	ND^d^
	All	49	2.52 × 10^8^	5.65 × 10^8^	1.19 × 10^9^	2.50 × 10^7^	2.85 × 10^4^
South Shore WWTP	Composite	33	2.77 × 10^8^	5.51 × 10^8^	9.8 × 10^8^	3.02 × 10^7^	3.49 × 10^4^
	Single	16	3.70 × 10^8^	8.08 × 10^8e^	7.89 × 10^8^	3.46 × 10^7^	ND
	All	49	3.04 × 10^8^	6.2 × 10^8^	9.13 × 10^8^	3.16 × 10^7^	3.49 × 10^4^
Both WWTPs	All ± SD^f^	98	2.77 × 10^8^ ± 1.74 × 10^8^	5.94 × 10^8^ ± 1.70 × 10^8^	1.04 × 10^9^ ± 1.95 × 10^8^	2.81 × 10^7^ ± 1.58 × 10^8^	3.18 × 10^4^ ± 5.5 × 10^4^

a Seven-day composite samples and single-day samples from each wastewater treatment plant (WWTP).

b Copy numbers (CN) per liter (per liter to compare with norovirus).

c Genomic copies (GC) per liter.

d ND indicates not determined.

e Significantly higher than indicator concentrations in single South Shore WWTP samples (p < 0.05) compared to Jones Island WWTP composite (p < 0.05) and Jones Island WWTP single samples (p < 0.05); no other significant differences were noted.

f SD of log-transformed data, as data are displayed as geomeans.

**Table 2:** Comparison of two combined sewer overflow (CSO) events of different magnitudes. DOI: https://doi.org/10.1525/elementa.301.t2

Event	Duration (h)	Volume CSO (MG)^a^	Mean HB^b^ (CN^c^ 100 ml^−1^)	Mean sewage (%)^d^	Load^e^ (HB CN)
19 June 2009	38.5	935.7	2.26 × 10^5^	0.81	1.70 × 10^16^
20 June 2011	14.0	170.5	3.97 × 10^4^	0.14	2.74 × 10^15^

a Million gallons.

b Mean HB (human *Bacteroides*) concentration at estuary station during the CSO event.

c Copy number.

d Computed using a geomean of 2.77 × 10^7^ HB 100 ml^−1^ in untreated sewage.

e Based on flow measurements from USGS station and 2-h concentrations of human *Bacteroides* (HB); loads during the CSO event are displayed and loads for full event are shown in Table 3.

**Table 3:** Peak 24-h mean concentrations and event loads under various hydrological conditions. DOI: https://doi.org/10.1525/elementa.301.t3

Start date^a^	Event type	Precipitation (cm prior 24 h)	Peak mean 24-h concentration of HB (CN 100 ml^−1^)^b^	Peak mean 24-h concentration of Lachno2 (CN 100 ml^−1^)	Load HB (CN)
19 June 2009	CSO	11.9	2.98 × 10^5^	3.35 × 10^5^	1.78 × 10^16^
7 July 2010	CSO	21.6^c^	2.68 × 10^4^	7.69 × 10^4^	ND
20 June 2011	CSO	10.2	2.36 × 10^4d^	1.01 × 10^5^	3.71 × 10^15^
8 June 2009	Rain	3.8^c^	6.88 × 10^3^	8.01 × 10^3^	3.45 × 10^14^
22 October 2009	Rain	5.3	9.72 × 10^4^	8.12 × 10^3^	6.49 × 10^14^
5 April 2010	Rain	7.1	8.18 × 10^4^	ND^e^	1.48 × 10^15^
23 April 2010	Rain	4.4	1.17 × 10^4^	ND	ND
10 May 2010	Rain	5.1	4.79 × 10^3^	1.14 × 10^4^	ND
27 July 2011	Rain	3.1^c^	1.89 × 10^3^	ND	ND
12 October 2011	Rain	1.4	1.59 × 10^3^	ND	ND
16 June 2009	Baseflow	0.4	7.20 × 10^2^	6.84 × 10^2^	ND
18 May 2010	Baseflow	0.0	2.62 × 10^2^	2.71 × 10^2^	ND
25 July 2011	Baseflow	0.0	8.27 × 10^2^	ND	ND

a Hydrographs with culture and qPCR data for indicators appear in Figures S3–S13 and S14–24, respectively.

b Copy numbers (CN) per liter.

c includes rainfall totals from 24 hours prior, for 23 July 2010 the main storm event was not sampled due to flooding.

d peak 12-h average; combined sewer overflow (CSO) event spanned 12 h.

e Not determined.
