# Supplementary material for: Sewage loading and microbial risk in urban waters of the Great Lakes
Source: Elementa (Wash D C). Author manuscript; Available in PMC 2018 Nov 1. (PMC6211557; doi:10.1525/elementa.301)

Figure S14. Fecal indicator bacteria at Milwaukee River at the Mouth and stream discharge at four Milwaukee River Watershed sites. [Menomonee River, Menomonee River at Wauwatosa; Kinnickinnic River, Kinnickinnic River at S. 11th Street at Milwaukee; lowess, lowess smoothing curve from 5 minute discharge data within a seiche−effected river channel; Lachno2, Lachnospiraceae human marker; Ruminant, *Bacteroides* Ruminant marker].

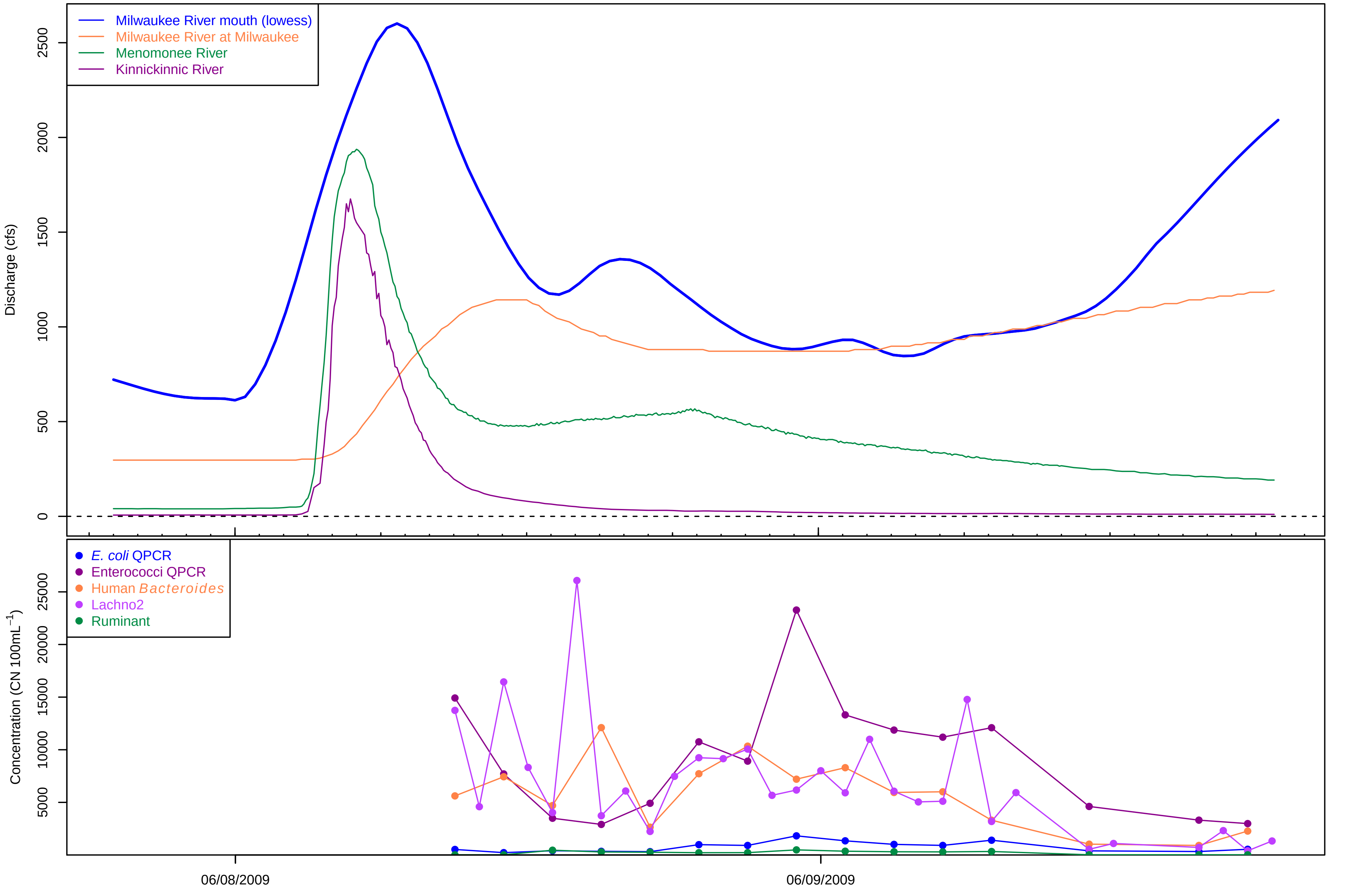

Figure S15. Fecal indicator bacteria at Milwaukee River at the Mouth and stream discharge at four Milwaukee River Watershed sites. [Menomonee River, Menomonee River at Wauwatosa; Kinnickinnic River, Kinnickinnic River at S. 11th Street at Milwaukee; lowess, lowess smoothing curve from 5 minute discharge data within a seiche−effected river channel; Lachno2, Lachnospiraceae human marker]

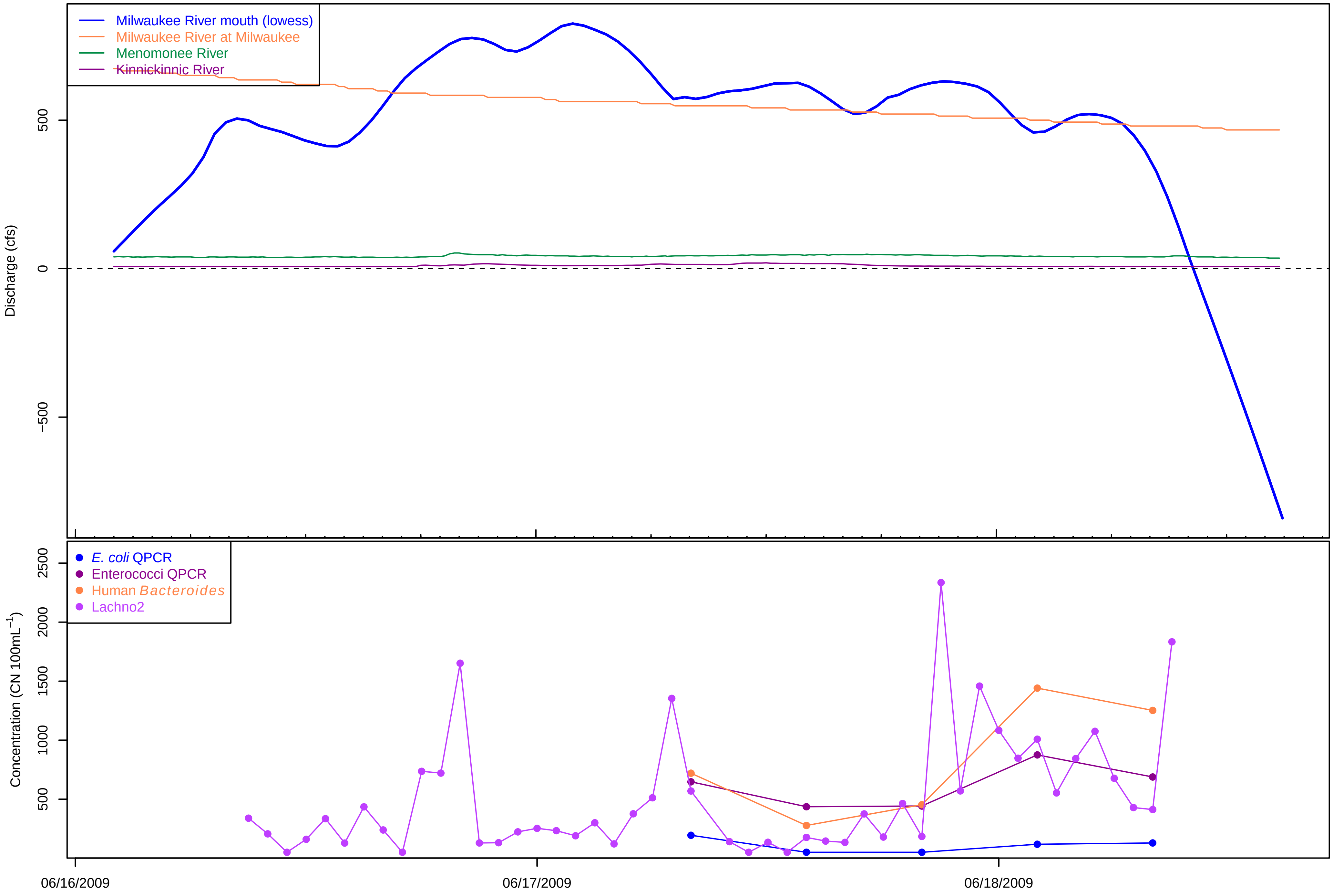

Figure S16. Fecal indicator bacteria at Milwaukee River at the Mouth and stream discharge at four Milwaukee River Watershed sites. [Menomonee River, Menomonee River at Wauwatosa; Kinnickinnic River, Kinnickinnic River at S. 11th Street at Milwaukee; lowess, lowess smoothing curve from 5 minute discharge data within a seiche−effected river channel; Lachno2, Lachnospiraceae human marker]

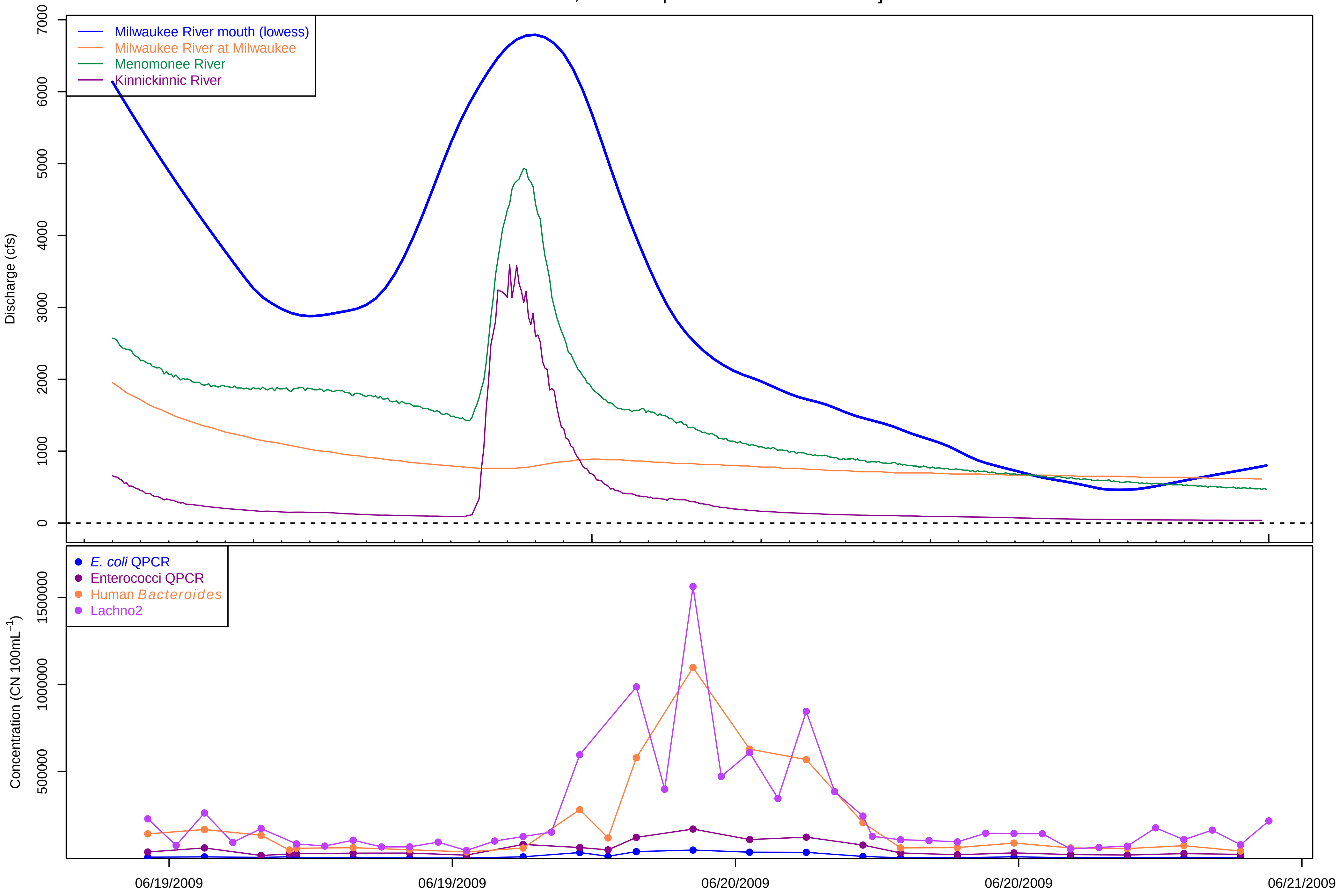

Figure S17. Fecal indicator bacteria at Milwaukee River at the Mouth and stream discharge at four Milwaukee River Watershed sites. [Menomonee River, Menomonee River at Wauwatosa; Kinnickinnic River, Kinnickinnic River at S. 11th Street at Milwaukee; lowess, lowess smoothing curve from 5 minute discharge data within a seiche−effected river channel; Lachno2, Lachnospiraceae human marker; Ruminant, *Bacteroides* Ruminant marker].

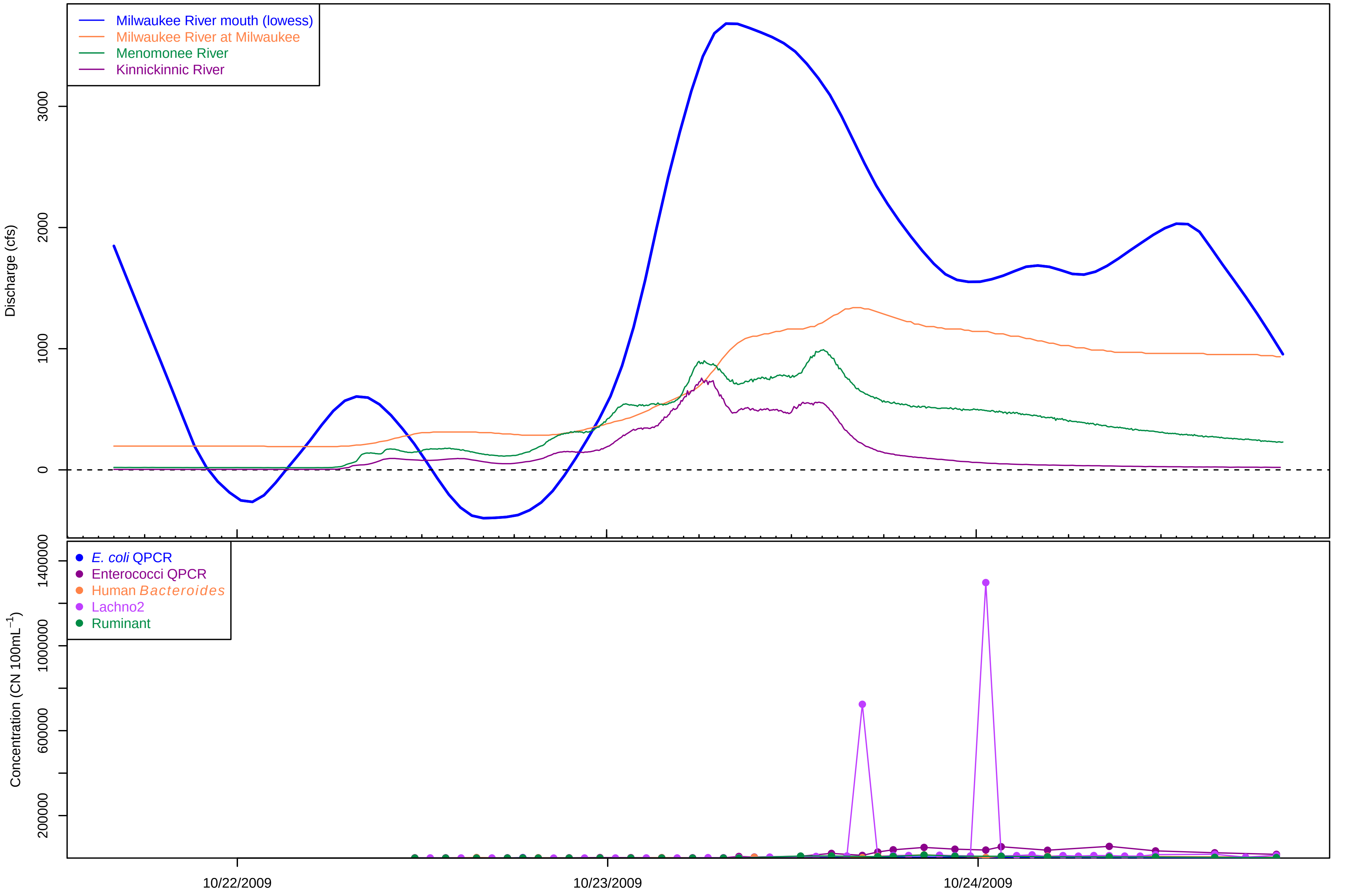

Figure S18. Fecal indicator bacteria at Milwaukee River at the Mouth and stream discharge at four Milwaukee River Watershed sites.
[Menomonee River, Menomonee River at Wauwatosa; Kinnickinnic River, Kinnickinnic River at S. 11th Street at Milwaukee;
lowess, lowess smoothing curve from 5 minute discharge data within a seiche−effected river channel;
Lachno2, Lachnospiraceae human marker]

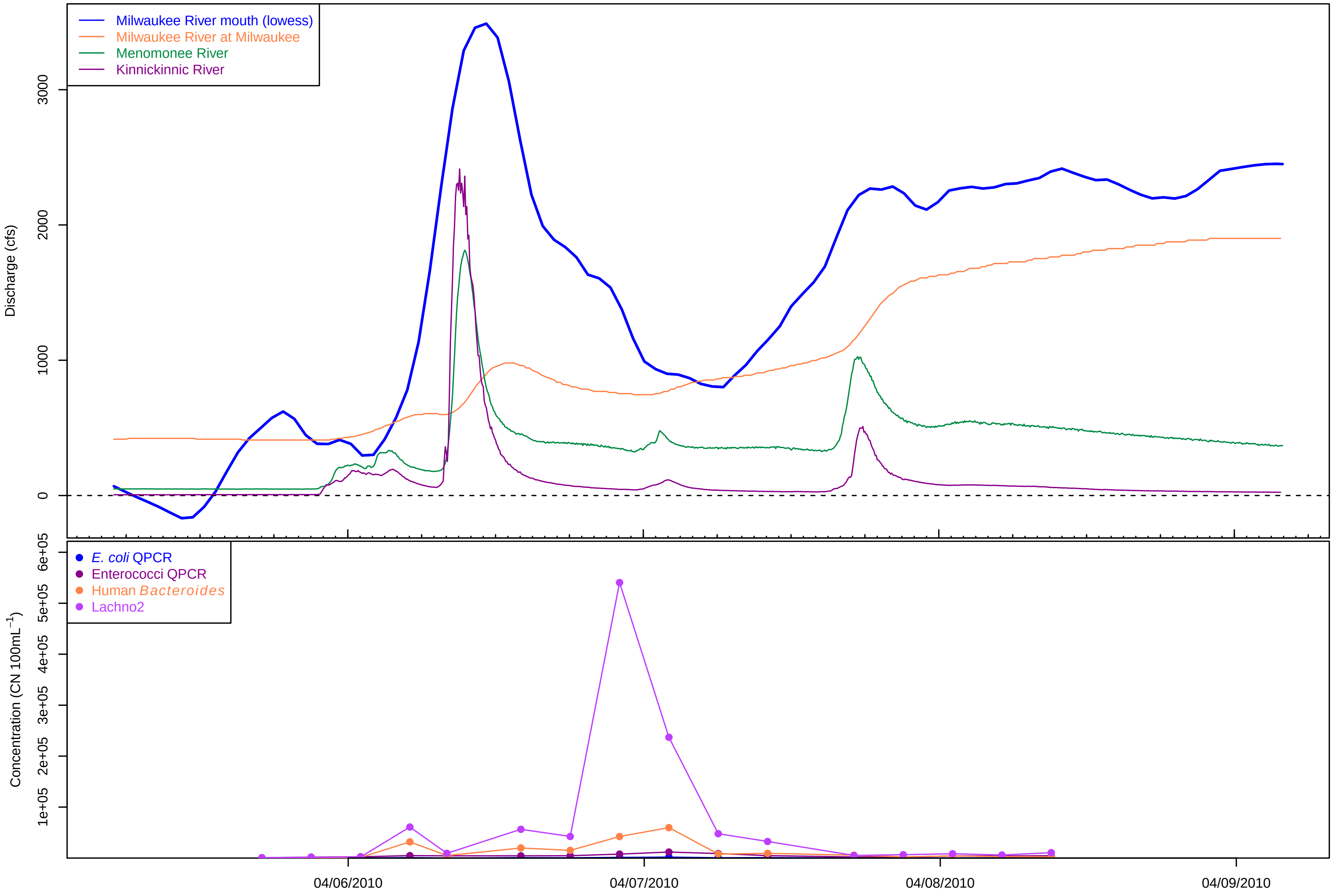

Figure S19. Fecal indicator bacteria at Milwaukee River at the Mouth and stream discharge at four Milwaukee River Watershed sites.
[Menomonee River, Menomonee River at Wauwatosa; Kinnickinnic River, Kinnickinnic River at S. 11th Street at Milwaukee;
lowess, lowess smoothing curve from 5 minute discharge data within a seiche−effected river channel;
Lachno2, Lachnospiraceae human marker]

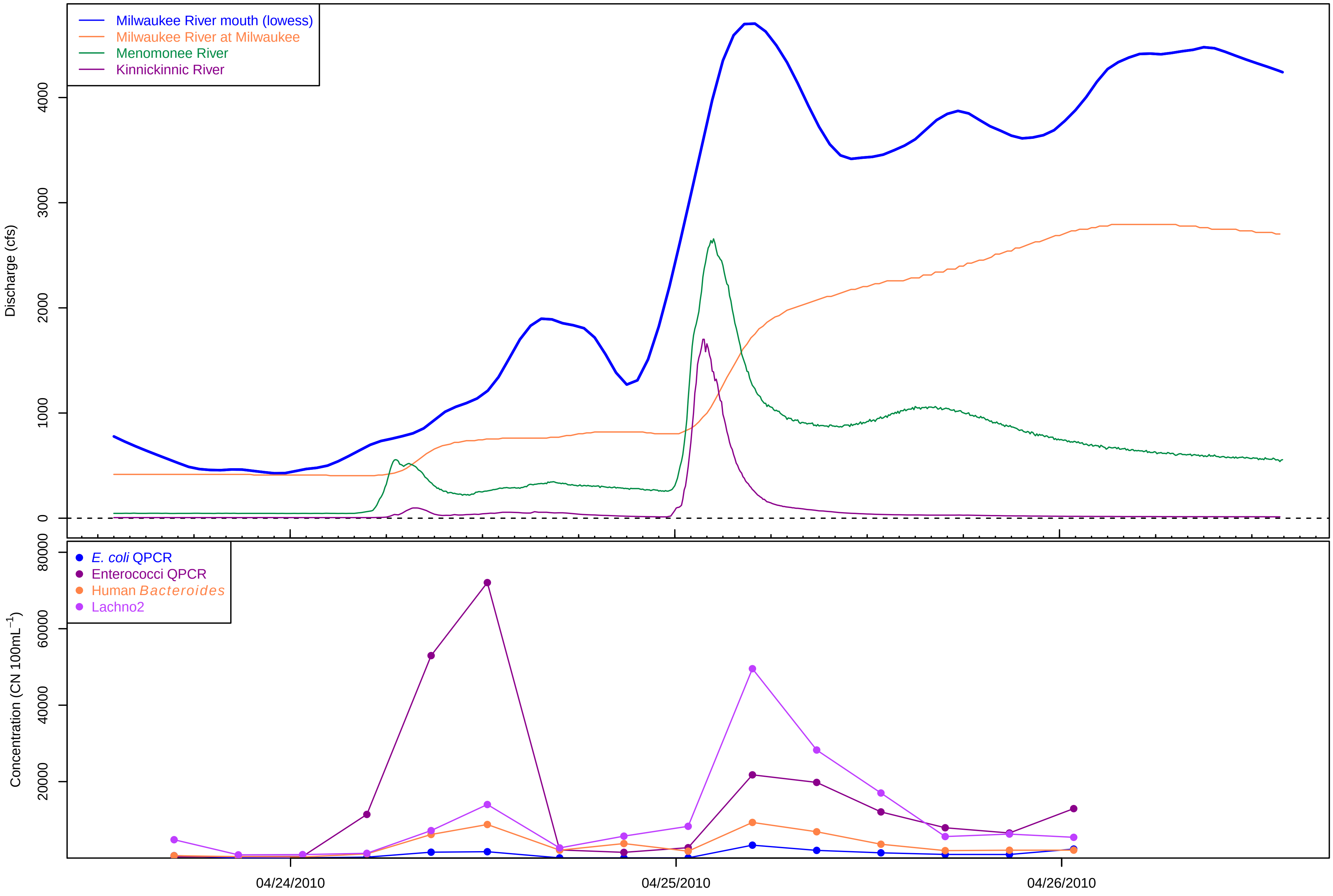

Figure S20. Fecal indicator bacteria at Milwaukee River at the Mouth and stream discharge at four Milwaukee River Watershed sites. [Menomonee River, Menomonee River at Wauwatosa; Kinnickinnic River, Kinnickinnic River at S. 11th Street at Milwaukee; lowess, lowess smoothing curve from 5 minute discharge data within a seiche−effected river channel; Lachno2, Lachnospiraceae human marker; Ruminant, *Bacteroides* Ruminant marker].

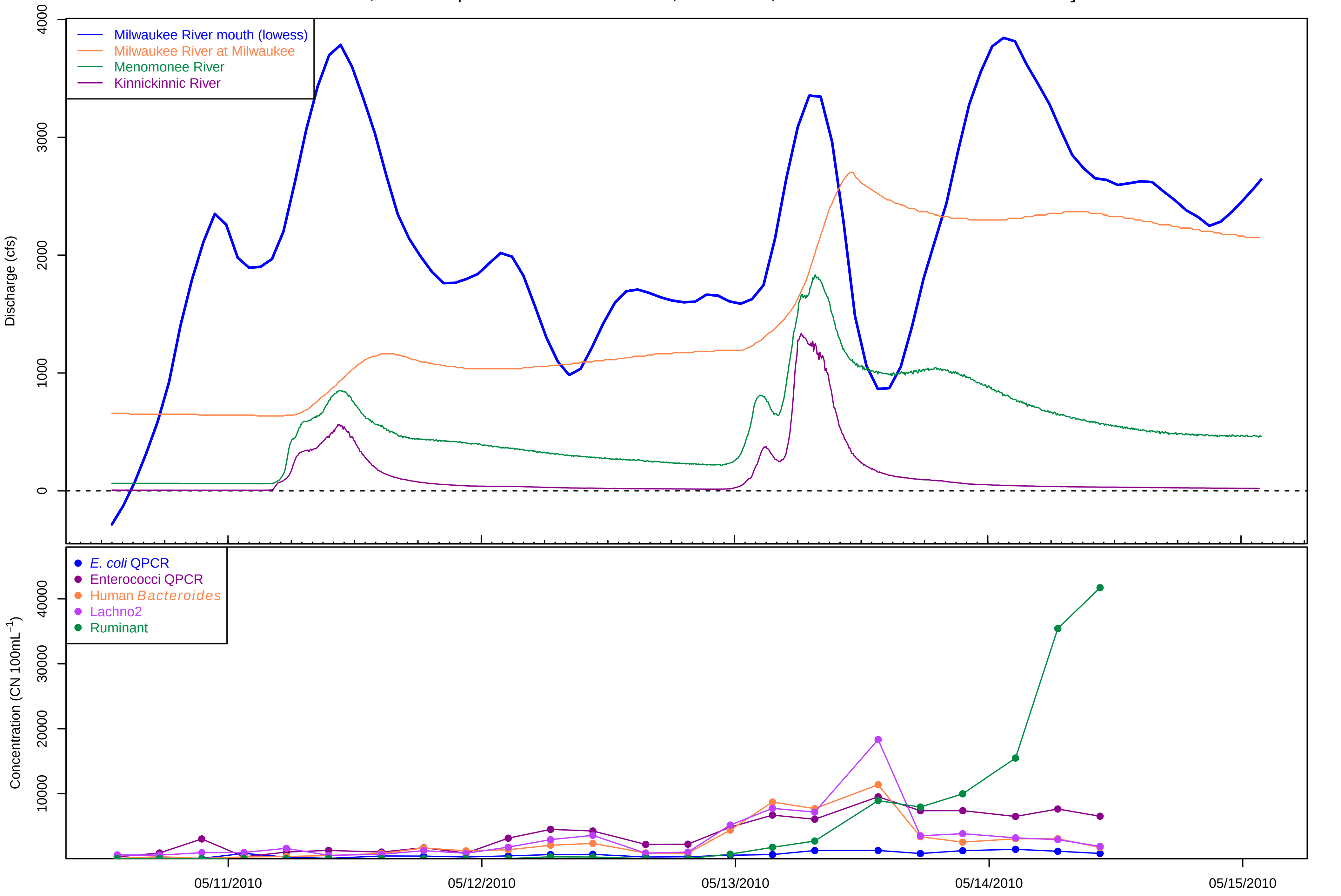

Figure S21. Fecal indicator bacteria at Milwaukee River at the Mouth and stream discharge at four Milwaukee River Watershed sites. [Menomonee River, Menomonee River at Wauwatosa; Kinnickinnic River, Kinnickinnic River at S. 11th Street at Milwaukee; lowess, lowess smoothing curve from 5 minute discharge data within a seiche−effected river channel; Lachno2, Lachnospiraceae human marker]

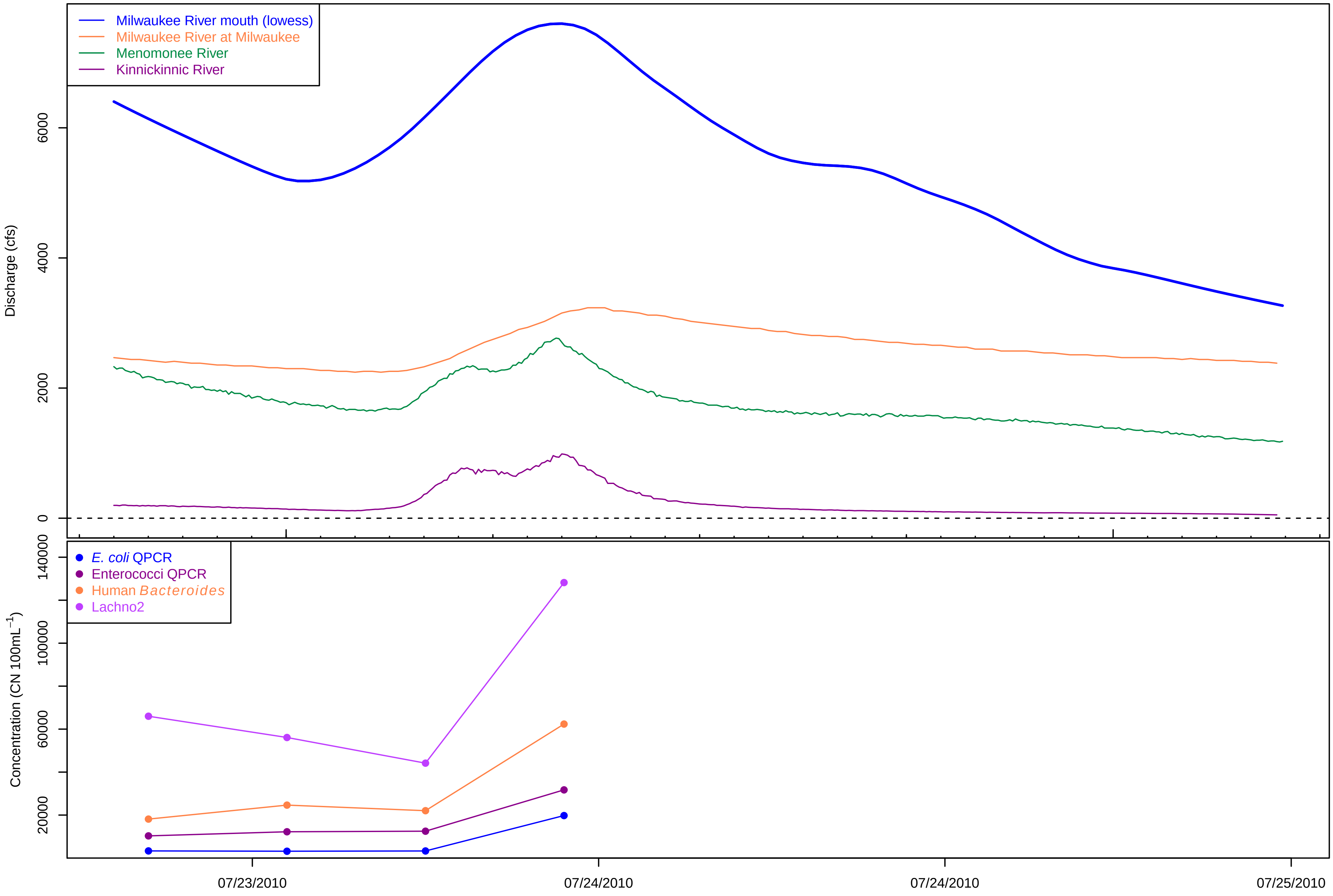

Figure S22. Fecal indicator bacteria at Milwaukee River at the Mouth and stream discharge at four Milwaukee River Watershed sites.
[Menomonee River, Menomonee River at Wauwatosa; Kinnickinnic River, Kinnickinnic River at S. 11th Street at Milwaukee;
lowess, lowess smoothing curve from 5 minute discharge data within a seiche−effected river channel;
Lachno2, Lachnospiraceae human marker]

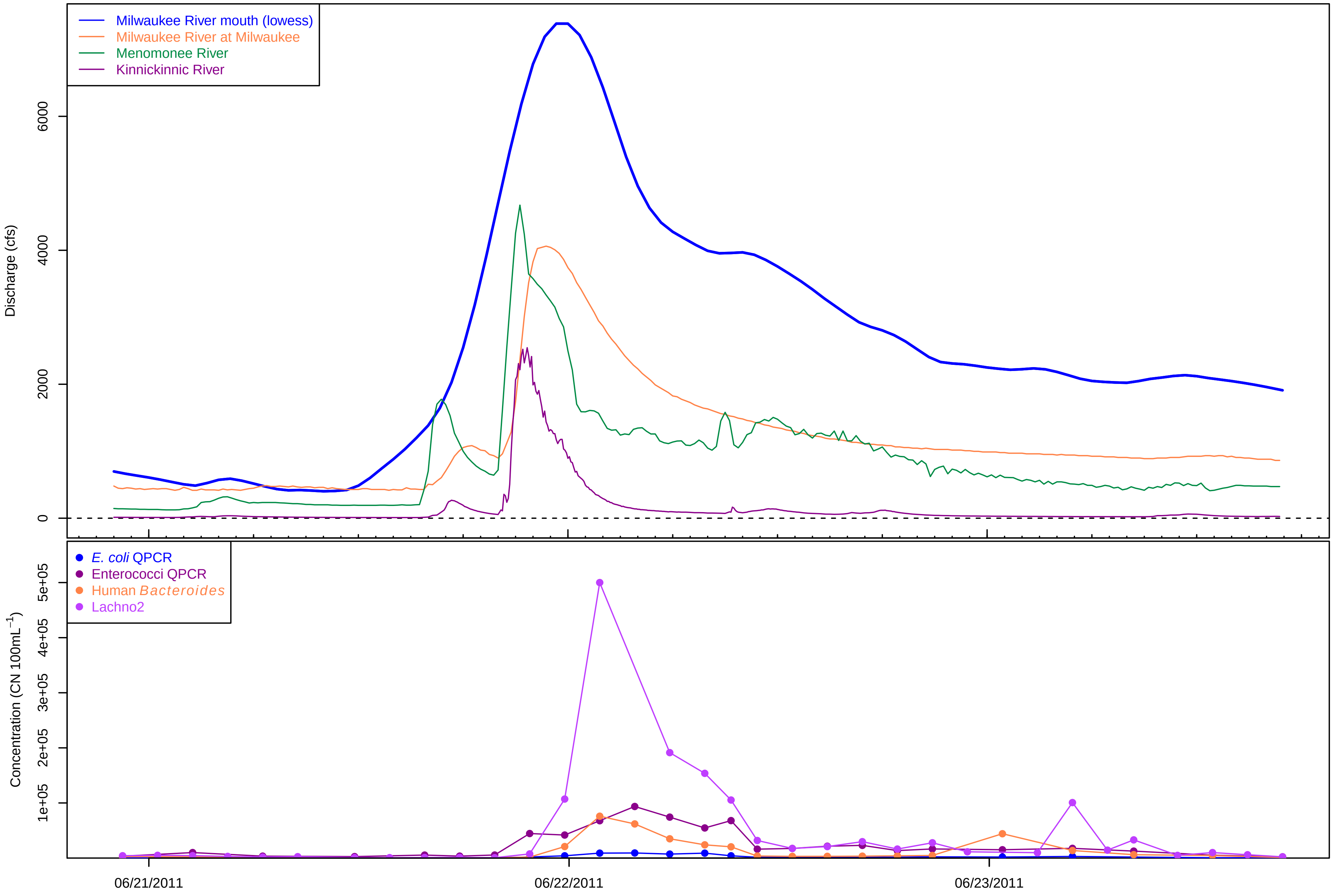

Figure S23. Fecal indicator bacteria at Milwaukee River at the Mouth and stream discharge at four Milwaukee River Watershed sites. [Menomonee River, Menomonee River at Wauwatosa; Kinnickinnic River, Kinnickinnic River at S. 11th Street at Milwaukee; lowess, lowess smoothing curve from 5 minute discharge data within a seiche−effected river channel; Lachno2, Lachnospiraceae human marker; Ruminant, *Bacteroides* Ruminant marker].

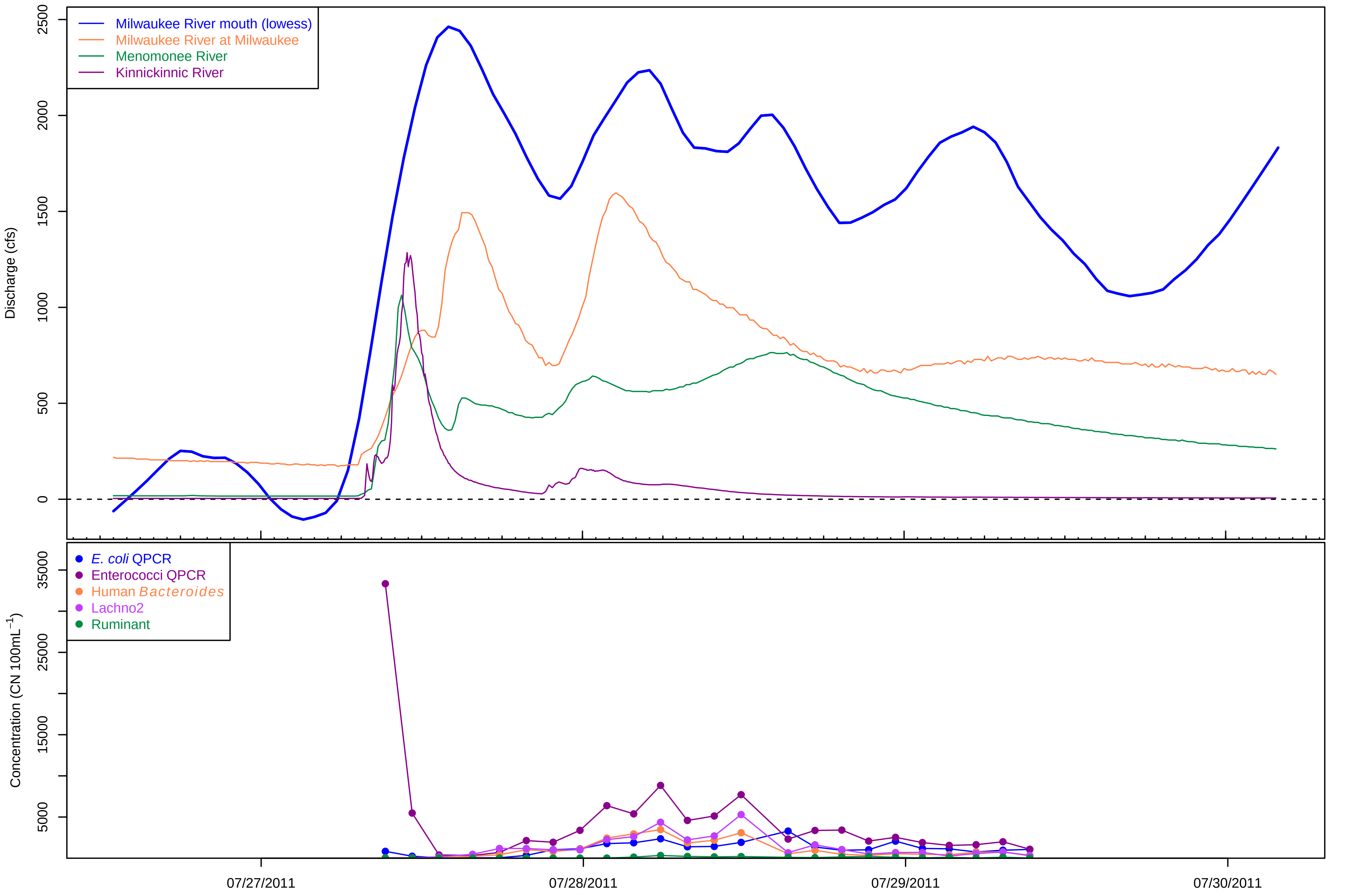

Figure S24. Fecal indicator bacteria at Milwaukee River at the Mouth and stream discharge at four Milwaukee River Watershed sites. [Menomonee River, Menomonee River at Wauwatosa; Kinnickinnic River, Kinnickinnic River at S. 11th Street at Milwaukee; lowess, lowess smoothing curve from 5 minute discharge data within a seiche−effected river channel; Lachno2, Lachnospiraceae human marker]

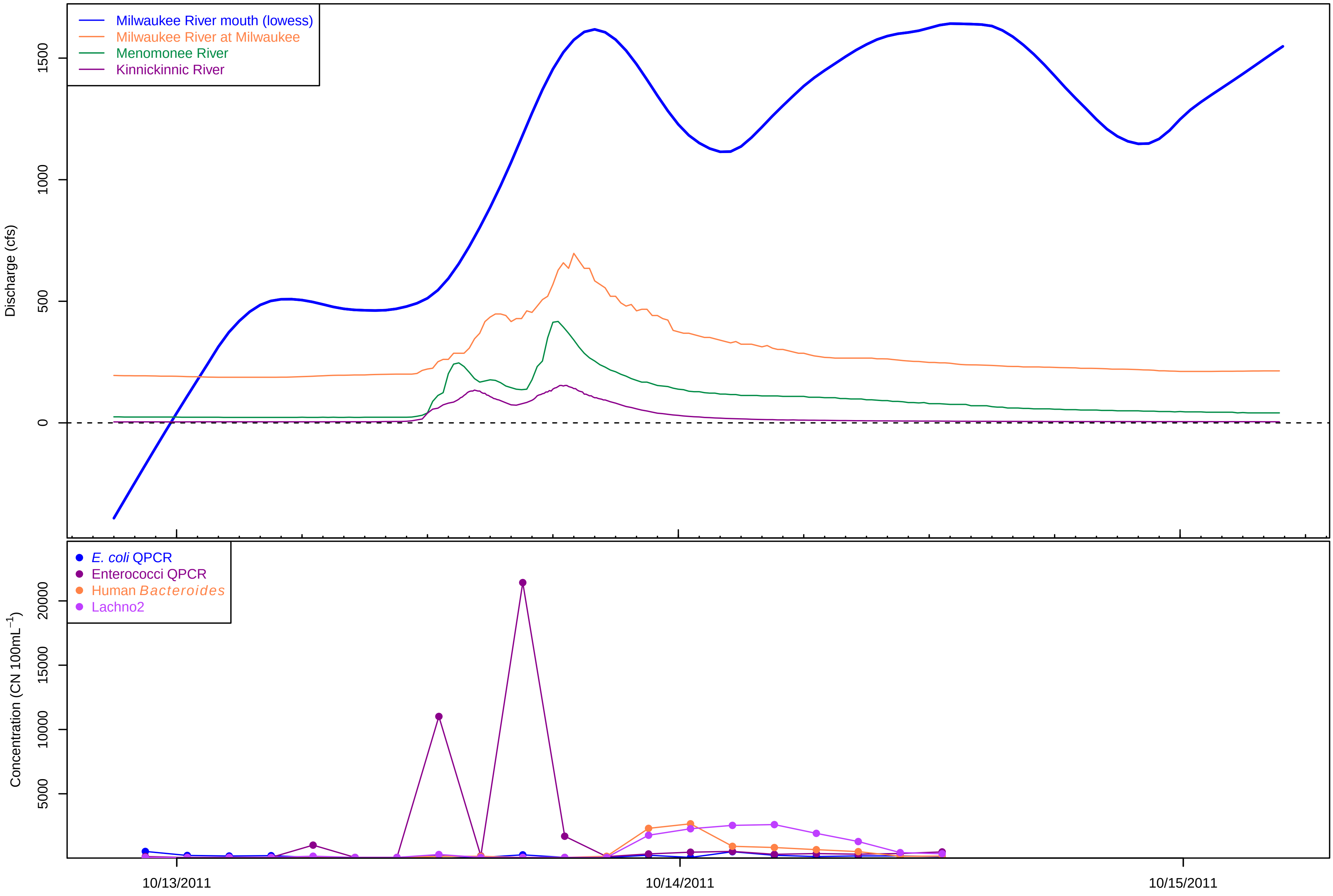

Supplement: Supplemental Fig S14-S24 [file NIHMS979489-supplement-Supplemental_Fig_S14-S24.pdf]
