# Supplementary material for: Sewage loading and microbial risk in urban waters of the Great Lakes
Source: Elementa (Wash D C). Author manuscript; Available in PMC 2018 Nov 1. (PMC6211557; doi:10.1525/elementa.301)
Supplement: Supplemental Text_Tables_Fig S1-S2 [file NIHMS979489-supplement-Supplemental_Text_Tables_Fig_S1-S2.pdf]

Supplemental material

Sewage loading and microbial risk in urban waters of the Great Lakes

Sandra L. McLellan[1*,] Elizabeth P. Sauer[1], Steve R. Corsi[2], Melinda J. Bootsma[1], Alexandria B. Boehm[3], Susan K. Spencer[4], Mark A. Borchardt[4]

[1]School of Freshwater Sciences, University of Wisconsin-Milwaukee, Milwaukee, Wisconsin, [2]United States Geological Survey, Middleton Wisconsin. [3]Department of Civil Engineering, Stanford University, Palo Alto, California, and [4]United States Department of Agriculture – Agricultural Research Service, Marshfield, WI.

Corresponding authors email: mclellan@uwm.edu

Disclaimer: Any use of trade, firm, or product names is for descriptive purposes only and does not imply endorsement by the U.S. Government.

List of contents:

Text S1. Detection of norovirus

Table S1. Urban estuary samples collected under low-flow, rainfall, and rainfall with combined sewer overflows (CSOs)

Table S2. Traditional host-associated qPCR assay primers, standard curves, and references

Table 3S. Log_{10} mean and standard deviation (in log space) of HB, Lachno2, and norovirus

Figure S1. Box and whisker plots of risk of illness from norovirus given a concentration of HB in river water. Analysis assumes that source of HB is raw sewage that is recent; i.e., 0–7 days. The red line shows the threshold illness level of 30/1000. The middle line in each box is the median probability of illness (P_{ill}) from the 10,000 iterations of the model. The top and bottom of the boxes are the 75th and 25th percentiles, respectively. The top and bottom of each whisker are the 90th and 10th percentiles, respectively, of those distributions.

Figure S2. Box and whisker plots of risk of illness from norovirus given a concentration of Lachno2 in river water. Analysis assumes that source of Lachno2 is raw sewage that is recent; i.e., 0–7 days. The red line shows the threshold illness level of 30/1000. The middle line in each box is the median P_{ill} from the 10,000 iterations of the model. The top and bottom of the boxes are the 75th and 25th percentiles, respectively. The bottom and top of each whisker are the 90th and 10th percentiles, respectively, of those distributions.

Supplemental Text S1. Detection of norovirus

For analysis of untreated wastewater treatment plant influent, a volume of 2 L was concentrated by standard polyethylene glycol flocculation methods (Lambertini et al., 2008). Final concentrated sample volumes, typically 2 to 4 mL, were stored at –20°C until nucleic acid extraction. The QIAamp DNA blood mini kit and buffer AVL (Qiagen, Valencia, CA) were used to extract nucleic acids from these stored volumes.

Samples were analyzed by two-step reverse transcription (RT)-qPCR (RNA viruses) using SuperScript III (Life Technologies, Carlsbad, CA) and LightCycler 480 Probes Master kit (Roche Diagnostics, Mannheim, Germany). The RT qPCR procedures are described in detail by Borchardt et al. (2012). Primers targeting GI norovirus (Jothikumar et al., 2005) and GII norovirus (Kageyama et al., 2003) have been described previously. Primer (IDT, Coralville, IA) and hydrolysis probe (TIB Molbio, Berlin, Germany) concentrations were 250 nM and 50 nM, respectively. Efficiencies for GI norovirus ranged from 0.924 to 1.066 with an average R^2 of 0.990. All samples were tested for RT and PCR inhibition following the method of Gibson et al. (2012). No-template controls (i.e., negative controls) for the RT, PCR, and extraction steps were negative. The qPCR standard curves were generated from archived stocks (cultures or human stool specimens) of each norovirus type. Standards were prepared and enumerated following the methods described in the Supplemental Material of Borchardt et al. (2012).

The 95% limit of detection (95% LOD) has been estimated for adenovirus and enterovirus previously, although the filtration step during LOD estimation used dead-end ultrafiltration instead of glass wool filtration. The 95% LODs were 1.5 and 4.0 gene copies L^{-1}, respectively (Stokdyk et al., 2016). These LODs are for the full analytical process including losses from filter elution, secondary concentration, and nucleic acid extraction. As the 95% LOD gives the lowest concentration at which there is a 95% probability of detection, concentrations reported below it are less likely to be detected but are, nonetheless, true positives.

References for Text S1

Borchardt MA, Spencer SK, Kieke BA, Lambertini E, Loge FJ. 2012. Viruses in nondisinfected drinking water from municipal wells and community incidence of acute gastrointestinal illness. *Environ Health Perspect* **120**(9): 1272–1279.

Gibson KE, Schwab KJ, Spencer SK, Borchardt MA. 2012. Measuring and mitigating inhibition during quantitative real time PCR analysis of viral nucleic acid extracts from large-volume environmental water samples. *Water Res* **46**(13): 4281–4291.

Jothikumar N, Lowther JA, Henshilwood K, Lees DN, Hill VR, et al. 2005. Rapid and sensitive detection of noroviruses by using TaqMan-based one-step reverse transcription-PCR assays and application to naturally contaminated shellfish samples. *Appl Environ Microbiol* **71**(4): 1870–1875.

Kageyama T, Kojima S, Shinohara M, Uchida K, Fukushi S, et al. 2003. Broadly reactive and highly sensitive assay for Norwalk-like viruses based on real-time quantitative reverse transcription-PCR. *J Clinical Microbiol* **41**(4): 1548–1557.

Lambertini E, Spencer SK, Bertz PD, Loge FJ, Kieke BA, et al. 2008. Concentration of enteroviruses, adenoviruses, and noroviruses from drinking water by use of glass wool filters. *Applied Environ Microbiol* **74**(10): 2990–2996.

Stokdyk JP, Firnstahl AD, Spencer SK, Burch TR, Borchardt MA. 2016. Determining the 95% limit of detection for waterborne pathogen analyses from primary concentration to qPCR. *Water Res* **96**: 105–113.

Table S1. Urban estuary samples collected under low-flow, rainfall, and rainfall with combined sewer overflows (CSOs)

Start date	End date	Event type	Number of samples	Number of samples analyzed using qPCR	Composite type for qPCR analysis
8 June 2009	11 June 2009	Rain	69	22	1-h sample
16 June 2009	18 June 2009	Baseflow	48	5	1-h sample
19 June 2009	22 June 2009	CSO	75	32	1-h sample
22 October 2009	25 October 2009	Rain	58	28	1-h sample
5 April 2010	8 April 2010	Rain	16	16	4-h composite
23 April 2010	26 April 2010	Rain	15	15	4-h composite
10 May 2010	14 May 2010	Rain	23	23	4-h composite
18 May 2010	19 May 2010	Baseflow	5	5	4-h composite
23 July 2010	27 July 2010	CSO	11	24	4-h composite
20 June 2011	24 June 2011	CSO	48	31	2-h composite
25 July 2011	26 July 2011	Baseflow	12	5	2-h composite
27 July 2011	29 July 2011	Rain	36	24	2-h composite
12 October 2011	14 October 2011	Rain	24	24	2-h composite
**TOTAL**			**476**	**254**	

Table S2. Traditional host-associated qPCR assay primers, standard curves, and references

Assay, target, references	Primers	Sequence	Slope and y intercept	Efficiency
E. coli, *uidA gene*, Li et al. (2006)	uidA1663F<br>uidA1790R<br>uidA1729p	5'GCG ACC TCG CAA GGC ATA3'<br>5'GAT TCA TTG TTT GCC TCC CTG CTG CG3'<br>5'[6FAM]-TGCAGCAGAAAAGCCGCCGACTTCGG [MGB-NFQ] 3'	–3.34, 38.69	99.41
Enterococci, 23S rRNA gene, USEPA (2012)	Entero1F-G<br>Entero2R<br>Enterop	5'GAG AAA TTC CAA ACG AAC TTG3'<br>5'CAG TGC TCT ACC TCC ATC ATT3'<br>5'[6FAM]-TGGTTCTCTCCGAAATAGCTTTAGGGCTA[MGB-NFQ] 3'	–3.37, 39.55	98.13
HB, 16S rRNA gene of human *Bacteroides,* Templar et al. (2016), modified from Bernhard and Field (2000) and Kildare et al. (2007)	HF183F<br>HF241R<br>HF193p	5′ATC ATG AGT TCA CAT GTC CG3'<br>5'CGT TAC CCC GCC TAC TAT CTA ATG3'<br>5'[6FAM]-TCC GGT AGA CGA TGG GGA TGC GTT [MGB-NFQ] 3'	–3.30, 37.72	100.85
Lachno2, human *Lachnospiraceae* (genus *Blautia*), Newton et al. (2011)	Lachno2-F<br>Lachno2-R<br>Lachno2p	5'TTCGCAAGAATGAAACTCAAAG3'<br>5'AAGGAAAGATCCGGTTAAGGATC3'<br>5'[6FAM]-ACCAAGTCTTGACATCCG [MGB-NFQ] 3'	–3.38, 37.37	97.76
Ruminant-specific *Bacteroidetes,* Reischer et al. (2006)	RumBacR_f<br>RumBacR_r<br>RumBacR_p	5'GCG TAT CCA ACC TTC CCG3'<br>5'CAT CCC CAT CCG TTAC CG3'<br>5'[6FAM]-CTT CCG AAA GGG AGA TT [MGB-NFQ]3'	–3.51, 40.97	92.82

References for Table 2S

Bernhard AE, Field KG. 2000. A PCR assay to discriminate human and ruminant feces on the basis of host differences in *Bacteroides-Prevotella* genes encoding 16S rRNA. *Appl Environ Microbiol* **66**:4571–4574.

Kildare BJ, Leutenegger CM, McSwain BS, Bambic DG, Rajal VB, Wuertz S. 2007. 16S rRNA-based assays for quantitative detection of universal, human-, cow-, and dog-specific fecal *Bacteroidales*: A Bayesian approach. *Water Res* **41**:3701–3715.

Li J, McLellan SL, Ogawa S. 2006. Accumulation and fate of green fluorescent labeled *Escherichia coli* in laboratory-scale drinking water biofilters. *Water Res* **40**:3023–3028.

Newton RJ, Vandewalle JL, Borchardt MA, Gorelick MH, McLellan SL, 2011. *Lachnospiraceae* and *Bacteroidales* alternative fecal indicators reveal chronic human sewage contamination in an urban harbor. *Appl Environ Microbiol* **77**:6972–6981.

Reischer G, Kasper DC, Steinborn R, Mach RL, Farnleitner AH. 2006. Quantitative PCR method for sensitive detection of ruminant pollution in freshwater and evaluation of this method in alpine karstic regions. *Appl Environ Microbiol* **8**:5610–5614.

Templar HA, Dila DK, Bootsma MJ, Corsi SR, McLellan SL. 2016. Quantification of human-associated fecal indicators reveal sewage from urban watersheds as a source of pollution to Lake Michigan. *Water Res* **100**:556–567.

USEPA. 2012. Method 1611: Enterococci in water by TaqMan® quantitative polymerase chain reaction (qPCR) assay. Office of Water, Washington, DC. EPA-821-R-12-008.

Table 3S. Log_{10} mean and associated standard deviation (in log space) of HB, Lachno2, and norovirus

Target	log_{10} mean[a]	SD of log_{10}-transformed values
HB (CN L^{-1})	8.442	0.2403
Lachno2 (CN L^{-1})	8.774	0.2325
Norovirus (GC L^{-1})	4.502	0.7535

[a]Units are log_{10} (copies per liter). Only positive values were used for determining the norovirus distribution of QMRA. A Lilliefors test of normality indicates that HB, Lachno2, and norovirus distributions were log-normal at $\alpha = 0.1$.

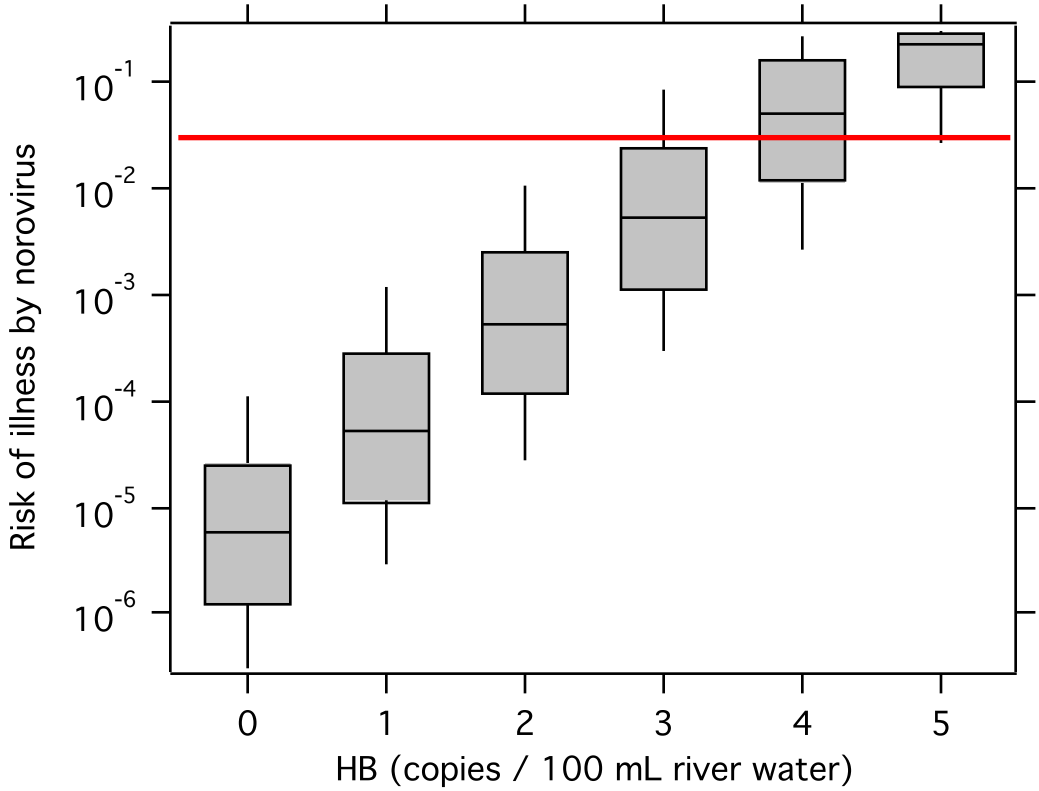

Figure S1. Box and whisker plots of risk of illness from norovirus given a concentration of HB in river water. Analysis assumes that source of HB is raw sewage that is recent; i.e., 0–7 days. The red line shows the threshold illness level of 30/1000. The middle line in each box is the median probability of illness (P_{ill}) from the 10,000 iterations of the model. The top and bottom of the boxes are the 75th and 25th percentiles, respectively. The top and bottom of each whisker are the 90th and 10th percentiles, respectively, of those distributions.

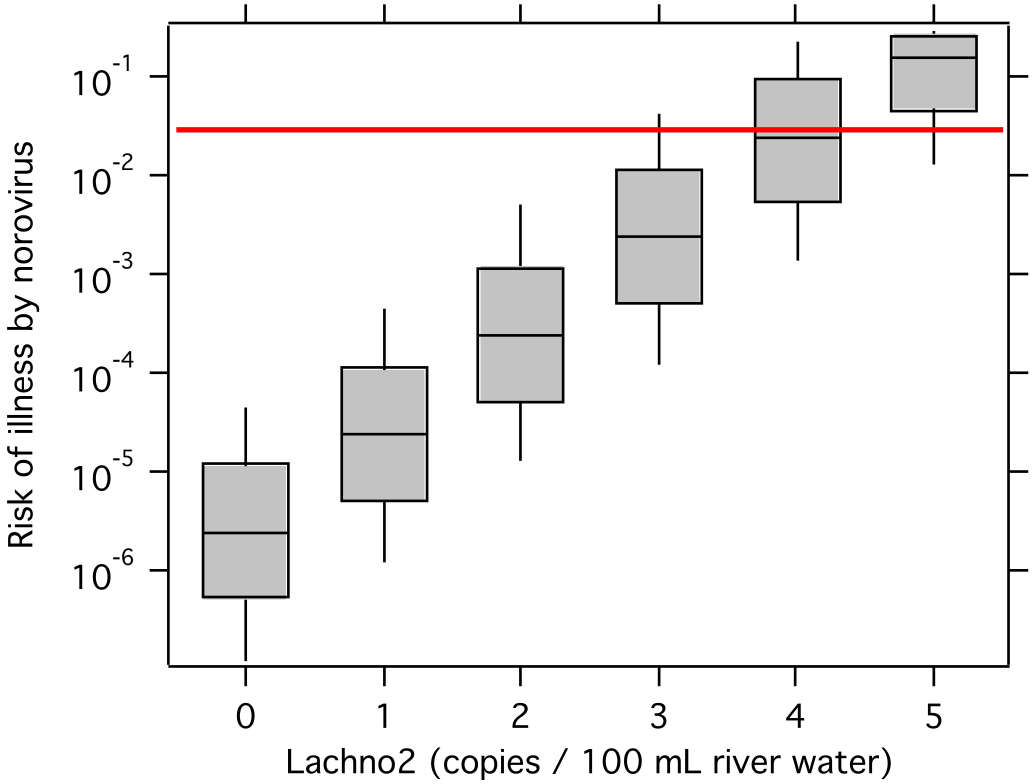

Figure S2. Box and whisker plots of risk of illness from norovirus given a concentration of Lachno2 in river water. Analysis assumes that source of Lachno2 is raw sewage that is recent; i.e., 0–7 days. The red line shows the threshold illness level of 30/1000. The middle line in each box is the median P_{ill} from the 10,000 iterations of the model. The top and bottom of the boxes are the 75th and 25th percentiles, respectively. The bottom and top of each whisker are the 90th and 10th percentiles, respectively, of those distributions.
